# OMG! A proteomic determinant of neurodegenerative resiliency

**DOI:** 10.1186/s13024-025-00921-1

**Published:** 2026-01-05

**Authors:** Michael R. Duggan, Hamilton Se-Hwee Oh, Philipp Frank, Gabriela T. Gomez, David Zweibaum, Yuhan Cui, Jingsha Chen, Aditya Surapaneni, Cassandra O. Blew, Heather E. Dark, Cassandra M. Joynes, Sridhar Kandala, Murat Bilgel, Amelia Farinas, Guray Erus, Qu Tian, Julián Candia, Krishna A. Pucha, Bennett A. Landman, Logan Dumitrescu, Timothy J. Hohman, Alexandria Lewis, Abhay Moghekar, Fatemeh Siavoshi, Muhammad Ali, Menghan Liu, Ying Xu, Daniel Western, Naoto Kaneko, Shintaro Kato, Makio Furuichi, Masaki Shibayama, Masahisa Katsuno, Yukiko Nishita, Rei Otsuka, Rebecca F. Gottesman, Eric B. Dammer, Nicholas T. Seyfried, Allan I. Levey, Erik C. B. Johnson, Elizabeth Mormino, Anthony D. Wagner, Kathleen L. Poston, Dimitrios Kapogiannis, Morgan E. Grams, Pavan Bhargava, Iwao Waga, Christos Davatzikos, Susan M. Resnick, Luigi Ferrucci, David A. Bennett, Carlos Cruchaga, Tony Wyss-Coray, Mika Kivimäki, Josef Coresh, Keenan A. Walker

**Affiliations:** 1 Laboratory of Behavioral Neuroscience, National Institute on Aging, NIH BRC BG RM 04B311, 251 Bayview Blvd, Baltimore, MD 21224 USA; 2 Graduate Program in Stem Cell and Regenerative Medicine, Stanford University, Stanford, CA USA; 3 The Phil and Penny Knight Initiative for Brain Resilience, Stanford University, Stanford, CA USA; 4 Wu Tsai Neurosciences Institute, Stanford University, Stanford, CA USA; 5 Department of Epidemiology and Public Health, University College London, London, UK; 6 Brain Sciences, University College London, London, UK; 7 Department of Medicine, Brigham and Women’s Hospital, Boston, MA USA; 8 Translational Gerontology Branch, National Institute on Aging, Baltimore, MD USA; 9 Artificial Intelligence in Biomedical Imaging Laboratory, Perelman School of Medicine, Philadelphia, PA USA; 10 Department of Epidemiology, Johns Hopkins Bloomberg School of Public Health, Baltimore, MD USA; 11 Department of Medicine, NYU Grossman School of Medicine, New York, NY USA; 12 Laboratory of Clinical Investigation, National Institute on Aging, National Institutes of Health, Baltimore, MD USA; 13 Department of Radiology & Radiological Sciences, Vanderbilt University Medical Center, Nashville, TN USA; 14 Vanderbilt University Institute of Imaging Science, Vanderbilt University Medical Center, Nashville, TN USA; 15 Department of Electrical and Computer Engineering, Vanderbilt University, Nashville, TN USA; 16 Department of Computer Science, Vanderbilt University, Nashville, TN USA; 17 Vanderbilt Memory and Alzheimer’s Center, Vanderbilt University Medical Center, Nashville, TN USA; 18 Vanderbilt Genetics Institute, Vanderbilt University Medical Center, Nashville, TN USA; 19 Department of Neurology, Johns Hopkins University School of Medicine, Baltimore, MD USA; 20 Department of Psychiatry, Washington University, St. Louis, MO USA; 21 NeuroGenomics and Informatics Center, Washington University, St. Louis, MO USA; 22 NEC Solution Innovators Limited, Koto-ku, Tokyo, Japan; 23 Foneslife Corporation, Chuo-ku, Tokyo, Japan; 24 Department of Neurology, Nagoya University Graduate School of Medicine, Nagoya, Aichi Japan; 25 Department of Clinical Research Education, Nagoya University Graduate School of Medicine, Nagoya, Aichi Japan; 26 Department of Epidemiology of Aging, National Center for Geriatrics and Gerontology, Obu, Aichi Japan; 27 Stroke Branch, National Institute of Neurological Disorders and Stroke, Bethesda, MD USA; 28 Department of Biochemistry, Emory University School of Medicine, Atlanta, Georgia USA; 29 Goizueta Brain Health Institute and Alzheimer’s Disease Research Center, Emory University School of Medicine, Atlanta, Georgia USA; 30 Center for Neurodegenerative Disease Center, Emory University School of Medicine, Atlanta, Georgia USA; 31 Department of Neurology, School of Medicine, Emory University, Atlanta, Georgia USA; 32 Department of Neurology and Neurological Sciences, Stanford University School of Medicine, Stanford, CA USA; 33 Department of Psychology, Stanford University, Stanford, CA USA; 34 Tohoku University, Aoba-ku, Sendai, Japan; 35 Rush Alzheimer’s Disease Center, Rush University Medical Center, Chicago, IL USA; 36 Department of Neurology, Washington University School of Medicine, St. Louis, MO USA; 37 University of Helsinki, Helsinki, Finland; 38 Department of Population Health, NYU Grossman School of Medicine, New York, NY USA

## Abstract

**Background:**

Biofluid proteomics can enhance our understanding of the neurodegenerative mechanisms underlying Alzheimer’s disease and related dementias (ADRDs). Oligodendrocyte myelin glycoprotein (OMG) is a brain-specific protein implicated in myelination, but its potential mechanistic, biomarker, and therapeutic roles in ADRDs requires further elucidation.

**Methods:**

After detecting an inverse association between its abundance in peripheral circulation and cortical amyloid deposition in two community-based cohorts, the current study characterized OMG’s role in ADRDs with high-throughput proteomics from sixteen independent cohorts. Data included a variety of cross-sectional and longitudinal community-based and clinical cohorts from North America, Europe, and Asia, and incorporated complementary biofluids, biospecimens, and proteomic platforms. Statistical analyses were conducted separately in each cohort.

**Results:**

We detected lower plasma OMG in individuals with cortical amyloid deposition, compromised brain structure, dementia, and multiple sclerosis, as well as in individuals who developed dementia over 7- to 20-year follow-up periods. OMG’s CSF and brain proteomic signatures reflected broader neuroprotective mechanisms, especially axonal structural integrity, and two-sample Mendelian randomization causally implicated OMG as protective against multiple neurodegenerative diseases.

**Conclusions:**

Our findings implicate OMG as a mechanistic determinant of neurodegenerative resiliency among older adults, which is reliably captured by its abundance in peripheral circulation

**Supplementary Information:**

The online version contains supplementary material available at 10.1186/s13024-025-00921-1.

## Introduction

Despite recent advances, we lack a full understanding of the neurodegenerative mechanisms underlying Alzheimer’s disease (AD) and related dementias (ADRDs). Cortical amyloid-β (Aβ) deposition is an early hallmark of AD, and AD accounts for the majority of dementia diagnoses, but individuals with dementia most commonly exhibit multiple neuropathological features (e.g., tauopathy, cerebrovasculopathy, synucleinopathy, etc.,) [1, 2] Furthermore, less than half of the variation in cognitive decline is explained by Aβ, neurofibrillary tangles, and other known neuropathologies [3]. Because proteins in peripheral circulation can originate from the brain and interact with CNS cell types, biofluid proteomics (e.g., SomaScan™ assay) can reveal scalable, readily accessible predictors and drivers of dementia and other age-related neurological outcomes [4–6]. Identifying proteomic mediators that reliably predict complementary neurodegenerative phenotypes across independent cohorts may unlock cost-effective biomarkers to detect individuals who can benefit from therapeutic interventions, guide the development of protein-targeting therapeutics, and illuminate neurobiological processes that afford resiliency against neurodegeneration.

After identifying oligodendrocyte myelin glycoprotein (OMG) as a CNS-specific protein whose levels in peripheral circulation were inversely associated with cortical Aβ deposition in two community-based cohorts, the current study leveraged high-throughput plasma proteomic data from over a dozen independent cohorts to characterize OMG’s role in ADRDs [7–12]. We found lower plasma OMG levels among individuals with dementia, compromised brain structure (measured with 3T MRI), and multiple sclerosis (MS). Additionally, individuals with lower plasma OMG were at elevated risk for future dementia and faster cognitive decline. Using its multi-cohort, CSF proteomic signature, we demonstrated that higher OMG abundance is reflective of broader neuronal and oligodendroglial mechanisms that primarily promote the maintenance of axonal structural stability, along with cell adhesion, synaptic functioning, and proteostasis. Having identified similar structural- and axonal-integrity pathways in OMG’s conserved brain tissue proteomic signature, we used genetic inference techniques to show that the cis regulation of OMG abundance across biofluids and brain tissue is causally implicated as protective against multiple neurodegenerative diseases.

## Methods

Below are general descriptions of the cohorts and primary outcomes used in the current study. Detailed descriptions and supporting references are provided in the Supplementary Methods. To ensure results were comparable to existing and future reports, the data processing, quality control, and analytic approaches followed established protocols and standardized guidelines in each cohort. The manuscript follows Strengthening the Reporting of Observational Studies in Epidemiology guidelines.

### Study sample

**Fig. 1:**
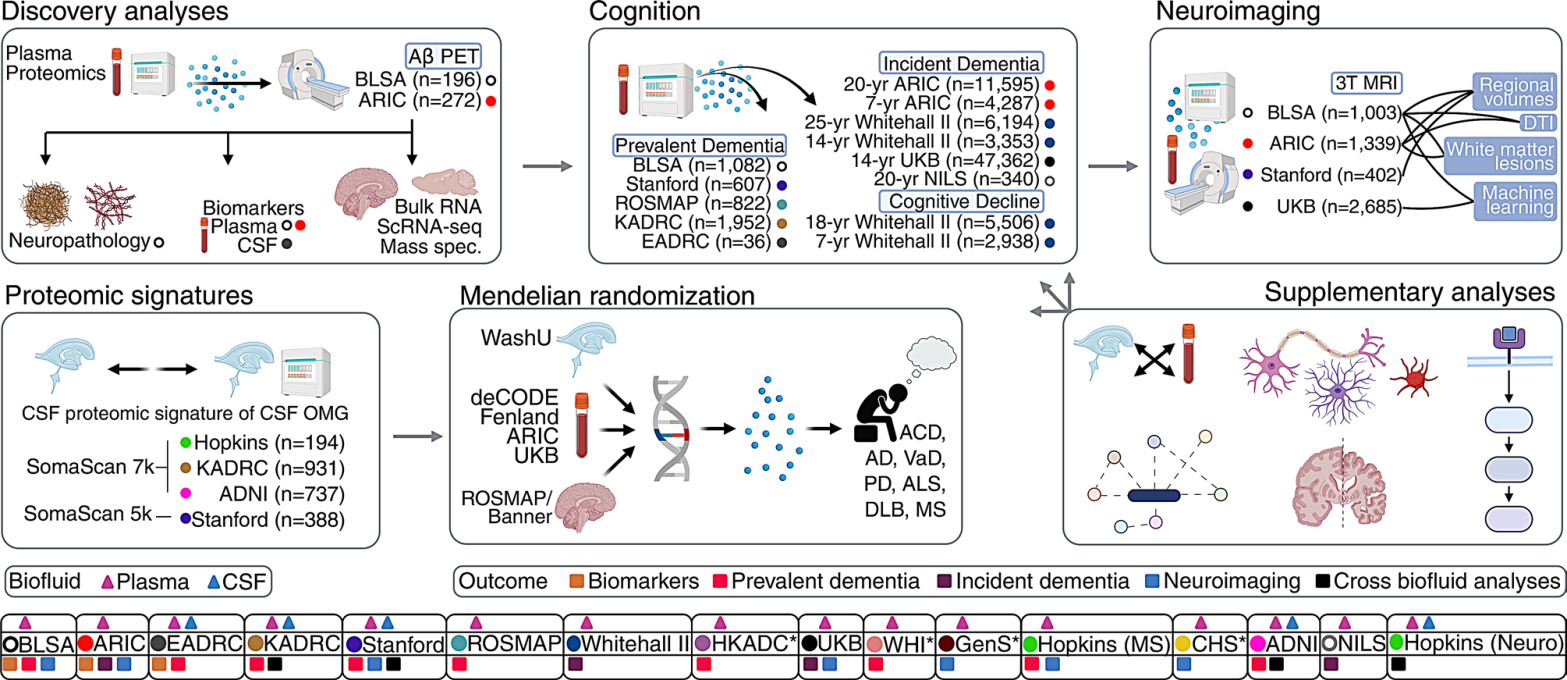
Study overview. The current study leveraged proteomics from the Baltimore Longitudinal study of Aging (BLSA), the Atherosclerosis risk in Communities study (ARIC), the Emory AD Research Center (Emory-ADRC; EADRC), a Stanford University cohort (i.e., participants enrolled in the Iqbal Farrukh and Asad Jamal Stanford ad Research Center, the Stanford Aging and Memory study, the Stanford Biomarkers in PD study, and the Stanford Center for Memory Disorders cohort study), the Religious Orders study/Rush Memory and Aging Project (ROSMAP), the Knight ad Research Center (Knight-ADRC; KADRC), a Hong Kong AD cohort (HKADC), the Women’s Health Initiative (WHI), the UK Biobank (UKB), the AD Neuroimaging Initiative (ADNI), the National Institute for Longevity Sciences-Longitudinal study of Aging (NILS-LSA), the Whitehall II cohort, the Cardiovascular Health study (CHS), the Generation Scotland study (GenS), the Johns Hopkins multiple sclerosis Center (JHMSC), and the Johns Hopkins Neurology cohort (JHN). *previously computed results were obtained for HKADC, WHI, CHS, and GenS. JHMSC analyses examined prevalent multiple sclerosis, not dementia

The current study leveraged proteomic data from multiple independent cohorts (Fig. 1), including the Baltimore Longitudinal Study of Aging (BLSA), the Atherosclerosis Risk in Communities study (ARIC), the Emory AD Research Center (Emory-ADRC), a Stanford University cohort (i.e., participants enrolled in the Iqbal Farrukh and Asad Jamal Stanford AD Research Center, the Stanford Aging and Memory Study, the Stanford Biomarkers in PD Study, and the Stanford Center for Memory Disorders Cohort Study), the Religious Orders Study/Rush Memory and Aging Project (ROSMAP), the Knight AD Research Center (Knight-ADRC), a Hong Kong AD cohort (HKADC), the Women’s Health Initiative (WHI), the UK Biobank (UKB), the AD Neuroimaging Initiative (ADNI), the National Institute for Longevity Sciences-Longitudinal Study of Aging (NILS-LSA), the Whitehall II cohort, the Cardiovascular Health Study (CHS), the Generation Scotland study (GenS), the Johns Hopkins Multiple Sclerosis Center (JHMSC), and the Johns Hopkins Neurology cohort (JHN).

### Proteomics

Proteomic analyses used data from the SomaScan platform for BLSA (v4.1, plasma), ARIC (v4.0, plasma), Emory-ADRC (v4.1, plasma, CSF), Stanford cohort (v4.1, plasma; v4.0, CSF), ROSMAP (v4.1, plasma), Knight-ADRC (v4.1, plasma, CSF), ADNI (v4.1, CSF), NILS-LSA (v4.1, plasma), Whitehall II (v4.1, v4.0, plasma), CHS (v4.0, plasma), GenS (v4.0, plasma), JHMSC (v4.1, plasma), and JHN (v4.1, plasma). Proteomic analyses used data from the Olink Explore platform for Emory-ADRC (plasma, CSF), HKADC (plasma), WHI (plasma), and UKB (plasma). Proteomic analyses used data from mass spectrometry for BLSA (brain tissue). Computed results were obtained for HKADC [7], WHI [9], CHS [10], and GenS [11]. Data were subjected to standardized quality control and established processing protocols in each cohort (e.g., sample exclusion based on manufacturer [SomaScan/Olink] recommendation, outlier detection and subsequent exclusion or winzorization, etc.,), and analyzed on a log_2_ scale unless otherwise noted. Intra-assay coefficients of variation (CV) estimate an assay’s precision and reliability [13]. Using 102 blind duplicates in the BLSA, OMG’s CV was 3.5%. Using 187 blind duplicates in ARIC, OMG’s intra-assay CV was 4.0% at Visit 2 and 6.6% at Visit 5.

### Imaging and biofluid biomarkers

Amyloid-beta (Aβ) PET analyses used data from BLSA (^11^C-Pittsburgh compound-B) and ARIC (^18^F-florbetapir). Aβ PET status (+/-) in BLSA was defined based on a Gaussian mixture model threshold of 1.064 mean cortical distribution volume ratio and in ARIC was defined based on a median of 1.20 mean cortical standard uptake value ratio. Plasma Aβ_40_, Aβ_42_, GFAP, NfL, pTau-181 analyses used data from BLSA (Simoa) and ARIC (Simoa), and CSF Aβ_42_, total tau, and pTau-181 analyses used data from Emory-ADRC (Luminex).

### Dementia ascertainment

Dementia status analyses used data from clinical adjudication (BLSA, ARIC, Emory-ADRC, Stanford, ROSMAP, Knight-ADRC, ADNI, HKADC, WHI), neuropsychological testing (NILS-LSA), or electronic health records (Whitehall II, UKB). Prevalent odds (BLSA, Emory-ADRC, Stanford, ROSMAP, Knight-ADRC, HKADC, WHI) and incident risk (ARIC, Whitehall II, UKB) were assessed. Etiology-specific associations were assessed where data were available (Whitehall II, UKB).

### Cognitive task performance

Cognitive performance analyses used cognitive domain data from BLSA (visuospatial ability, verbal memory, verbal fluency, executive functioning, attention, processing speed) and Whitehall II (executive functioning, memory, phonemic fluency, semantic fluency, global cognition). Scores were calculated from one or more individual task components. Cross-sectional differences (BLSA) and rates of change (Whitehall II) were assessed.

### MRI

Structural MRI analyses used 3T MRI data from BLSA, ARIC, GenS, UKB, and JHMSC; CHS used 1.5T MRI data. Regional volumes (BLSA, ARIC, GenS, JHMSC), white matter hyperintensities (BLSA, ARIC, GenS, CHS, JHMSC), diffusion tensor imaging (BLSA, ARIC, GenS), and machine-learning atrophy measures (BLSA, UKB, GenS) were assessed. Regional anatomic labeling of T1-weighted images used the Multi-atlas Region Segmentation Utilizing Ensembles (BLSA, UKB), FreeSurfer (ARIC, GenS, CHS), or the Multi-atlas Cortical Reconstruction using Implicit Surface Evolution (JHMSC). White matter hyperintensity data were derived from an automated segmentation pipeline (BLSA, ARIC, GenS, JHMSC) or standardized and validated neuroradiologist procedures (CHS). Diffusion tensor data captured tract-specific (BLSA) or composite (ARIC, GenS) fractional anisotropy (FA) and mean diffusivity (MD) indices. Machine-learning atrophy measures reflected age-related brain volume loss derived with algorithms that were pretrained and validated in external cohorts; algorithms leveraged a semi-supervised representation learning via generative adversarial network (BLSA, UKB) or a Gaussian processes regression (GenS).

### Two-sample Mendelian randomization (MR)

Two-sample MR analyses used cis protein quantitative trait loci (pQTL) derived from GWAS of protein levels conducted by the Washington University in St. Louis (WashU; SomaScan; CSF), deCODE (SomaScan; plasma), the Fenland study (SomaScan; plasma), ARIC (SomaScan; plasma), UKB (Olink; plasma), and ROSMAP/Banner (mass spectrometry; brain tissue). Mass spectrometry on the whole brain proteome used TMT labeling and off-line high pH fractionation. These GWAS were conducted on proteomic measurements expressed on log_10_ (WashU, CSF), log_2_ (ROSMAP/Banner, brain tissue; ARIC, plasma; UKB, plasma) or rank-based inverse normal transformation (deCODE, plasma; Fenland, plasma) scales. Outcomes leveraged GWAS summary statistics of AD, all-cause dementia (ACD), vascular dementia (VaD), Parkinson’s disease (PD), amyotrophic lateral sclerosis (ALS), Lewy body dementia (DLB), and multiple sclerosis (MS). For CSF pQTLs, because no variants were detected at genome-wide significance (i.e., *p* < 5.0 × 10^−8^), an alternative significance threshold (*p* < 5.0x10^−4^) was used; for brain and plasma pQTLs, genome-wide significance thresholds were employed. pQTLs were pruned to remove variants in linkage disequilibrium (*r*^2^ < 0.05; 10 Mb window). Due to the availability of a single genetic instrument, the Wald ratio estimate was considered for primary analyses, and testing for heterogeneity between causal estimates and horizontal pleiotropy assumptions was not applicable.

### Proteomic biological characterization

The biological relevance and functional implications of OMG and its proteomic signatures were assessed with several complementary bioinformatic tools. Canonical pathways and functions of OMG were identified with annotated terms in the Ingenuity Knowledge Base available through Ingenuity Pathway Analysis. Protein-protein interactions were identified with the Human Integrated Protein-Protein Interaction Reference database. Expression data were primarily obtained from the Genotype-Tissue Expression, Human Protein Atlas, Seattle Alzheimer’s Disease Brain Cell Atlas, and Accelerating Medicines Partnership Program for AD databases. Enriched biological processes were identified with Ingenuity Pathway Analysis and the Enrichr platform. Supplementary information on genetic variants was obtained with the OpenTargets, Genecards, OnTime, and Genotype-Tissue Expression platforms.

### Statistical analyses

Statistical analyses were conducted separately in each cohort for each outcome of interest rather than conducting analyses on harmonized proteomic and outcome data across cohorts due to the inherently heterogenous study designs, data structures, and outcome distributions between cohorts. A hierarchical gating approach was employed, whereby proteins of interest for downstream analyses were first identified with discovery analyses of cortical Aβ deposition in two community-based cohorts (BLSA, ARIC). We subsequently employed multiple external cohorts and orthogonal strategies to support associations between OMG and ADRD endophenotypes (e.g., prevalent and incident cognitive impairment, biofluid biomarkers, structural neuroimaging etc.,). Covariates were selected based on theory-driven rationale and data availability. Participants with missing predictor, outcome, and/or covariate data were not included in analyses.

In the BLSA, logistic regression adjusted for age, sex, race, education, *APOE*ε4, and a comorbidity index was used to examine associations with Aβ PET and cognitive status. Multiple linear regression adjusted for the aforementioned covariates was used to examine associations with 3T MRI measures. Regional and voxel-wise brain volume analyses also adjusted for total intracranial volume, white matter volume analyses also adjusted for total white matter volume, and plasma biomarker analyses also adjusted for eGFR. Multiple linear regression adjusted for age, time between blood draw and autopsy, and most recent cognitive performance (Mini-Mental State Examination) was used to examine associations with neuropathology (Braak stages, CERAD scores) and mass spectrometry proteomics; time between blood draw and autopsy was adjusted for given the heterogeneity in duration between plasma and brain tissue sample collection dates. In ARIC, logistic regression adjusted for age, sex, race-center, education, *APOE*ε4, and cardiovascular risk factors (obesity, diabetes, hypertension) was used to examine associations with Aβ PET status. Plasma biomarker analyses also adjusted for eGFR, smoking status, and BMI instead of obesity. Cox proportional hazards regression adjusted for the aforementioned covariates was used to examine associations with incident dementia risk. For stratified analyses, cardiovascular disease was defined as the presence of one or more conditions (congestive heart failure, stroke, diabetes, ischemic heart disease), and high education was defined as having at least some level of college. Multiple linear regression adjusted for the aforementioned covariates plus intracranial volume was used to examine associations with 3T MRI measures. In the Emory-ADRC, logistic and linear regression adjusted for age, sex, and *APOE*ε4 were used to examine associations with cognitive status and CSF biomarkers, respectively. In the Stanford cohort, ROSMAP, Knight-ADRC, and ADNI, logistic regression adjusted for age, sex, and *APOE*ε4 was used to examine associations with cognitive status. Cohort specific effects were assessed in fixed effect inverse variance-weighted meta-analysis for primary dichotomous outcomes of interest (i.e., Aβ PET and cognitive status) and two-sample MR outcomes. Meta-analysis of cognitive status utilized odds ratios and error estimates of OMG derived only from SomaScan platform to avoid platform-related measurement heterogeneity. Heterogeneity statistics (Q, I^2^, t^2^) indicated no significant assumption violations and sensitivity analyses using random-effects models yielded similar results. In the Stanford cohort, regional brain volume analyses adjusted for age, sex, and total intracranial volume. In NILS-LSA, Cox proportional hazards regression adjusted for age, sex, and *APOE*ε4 was used to examine associations with incident dementia risk. In Whitehall II, Cox proportional hazards and linear mixed effects regression adjusted for age, sex, and ethnicity were used to examine associations with incident dementia risk and rates of cognitive decline, respectively. In the UKB, Cox proportional hazards regression adjusted for age, sex, education, study site, *APOE*ε4, eGFR, and cardiovascular risk factors (BMI, diabetes, hypertension, smoking) was used to examine associations with etiology-specific dementia risk. Multiple linear regression adjusted for age, sex, *APOE*ε4, eGFR, total household income, and cardiovascular risk factors (BMI, hypertension, diabetes, smoking) was used to examine associations with 3T MRI measures. In JHMSC, logistic and linear regression adjusted for age, sex, and race were used to examine associations with MS status and MS phenotypes, respectively; regional brain volume analyses also adjusted for total intracranial volume. For plasma-CSF proteomic associations in the Knight-ADRC, the Emory-ADRC, ADNI, and JHN, linear regression adjusted for age and sex were used.

Discovery analyses of cortical Aβ deposition used a nominally significant p-value of 0.05 (although FDR-corrected results are also presented) followed by the utilization of multiple external cohorts and orthogonal strategies to reduce the possibility of Type I error. Given the targeted nature of follow up ADRD endophenotype analyses (i.e., a single protein’s measurement [OMG] in relation to specific outcomes of interest [cognitive status, ADRD biomarkers, 3T MRI etc.,]), statistical significance for these analyses was defined as *p* < 0.05. Given the large number of comparisons when assessing OMG’s CSF, plasma, and brain proteomic signatures, statistical significance for these analyses was defined as FDR < 0.05. FDR corrections were applied per outcome within each cohort. Because the multiple testing burden, power, and outcome distributions can inherently differ by study design, harmonizing statistical thresholds across heterogeneous datasets was not employed. Regression analyses leveraged several commonly employed R packages, including stats and survival. Model quality and goodness of fit were assessed using the performance R package. Meta-analysis utilized the meta R package. Model assumptions were satisfied and normal distributions were verified. Proportional hazards assumptions were assessed with Kaplan Meier curves and Schoenfeld residuals. Linear regression equations followed standard formatting (i.e., outcome ~ predictor + covariates) and did not employ random effects for any included variables. Beta coefficients, odds ratios, and hazard ratios, were reported for linear, logistic, and Cox regressions, respectively. Analyses were performed using R (versions 4.2.2–4.4.1).

## Results

### Plasma OMG is associated with lower AD neuropathology

**Fig. 2:**
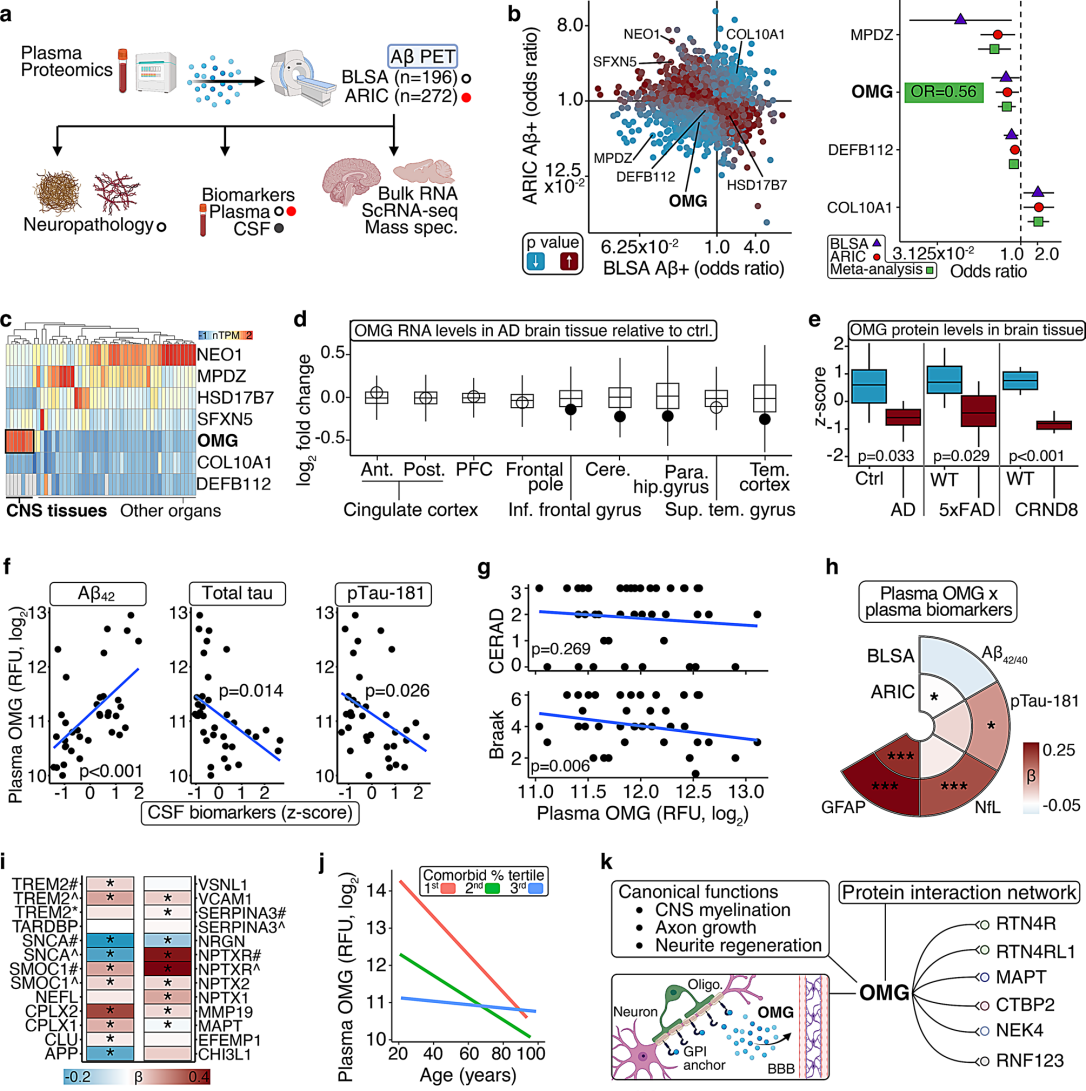
Plasma OMG is associated with lower AD neuropathology. **a**. Overview of discovery analyses. **b**. Plasma proteins associated with amyloid-beta (Aβ) pet positivity (+) in the BLSA (*n* = 196) and ARIC (*n* = 272). BLSA results derived from logistic regression models adjusting for age, sex, race, education, *APOE*ε4, and a comorbidity index. ARIC results derived from logistic regression adjusting for age, sex, race-center, education, *APOE*ε4, and cardiovascular risk factors. Proteins with significant associations across both cohorts are labeled. Meta-analyzed estimates for plasma proteins with directionally consistent relationships were derived from fixed effect inverse variance-weighted meta-analysis. Results are presented as odds ratios (ORs) and 95% confidence intervals (CIs). **c**. Expression levels across 54 tissue types based on bulk RNA sequencing (normalized transcripts per million; nTPM) from the human protein atlas. Dendrogram reflects hierarchical clustering using Euclidean distances. **d**. OMG regional differential expression in AD brain tissue relative to non-AD controls based on bulk RNA sequencing sourced from the Accelerating Medicines Partnership Program for AD. Black dots indicate significant (FDR *p* < 0.05) associations. Box plots: median, 25–75th quartiles; whiskers: 1.5× the IQR. **e**. OMG protein levels in AD-patient and -mouse brain tissue. Z-scores reflect frontal cortex levels in AD patients (*n* = 8) relative to controls (*n* = 8), hippocampal levels in 10.0-month old 5xFAD mice (*n* = 6) relative to wild type mice (*n* = 6), and total brain levels in 18.0-month old CRND8 mice (*n* = 4) relative to wild type mice (*n* = 4), respectively. Results derived from independent samples t-tests. Box plots: median, 25–75th quartiles; whiskers: 1.5× the IQR. **f**. CSF biomarkers associated with plasma OMG in the Emory-ADRC (*n* = 36). Results derived from linear regression adjusting for age, sex, and *APOE*ε4. **g**. Braak stages and CERAD scores associated with plasma OMG in the BLSA (*n* = 40). Results derived from linear regression adjusting for age, time between blood draw and autopsy, and most recent cognitive performance. **h**. Plasma biomarkers associated with plasma OMG in in the BLSA (Aβ_42/40_, NfL, GFAP *n* = 1,017; pTau181 *n* = 690) and ARIC (*n* = 1,299). BLSA results derived from linear regression adjusted age, sex, race, education, *APOE*ε4, eGFR, and a comorbidity index. ARIC results derived from linear regression adjusting for age, sex, race-center, education, *APOE*ε4, eGFR, and cardiovascular risk factors. ***indicates *p* < 0.001. *indicates *p* < 0.05. **i**. AD- and neurodegeneration-related plasma proteins associated with plasma OMG in the BLSA (*n* = 1,275). Results derived from linear regression adjusted age, sex, race, education, *APOE*ε4, and a comorbidity index. *indicates *p* < 0.05. **j**. Chorological age associated with plasma OMG in the BLSA (*n* = 1,275) stratified by comorbidity index tertile. Results derived from linear regression. **k**. Overview of OMG’s canonical functions and protein-protein interaction network. Amyloid-beta _42/40_ ratio, Aβ_42/40_; glial fibrillary acidic protein, GFAP; neurofilament light, NfL; phosphorylated tau 181, pTau-181; relative florescence units, RFU

To identify proteins in circulation associated with an early phase of AD, we conducted proteome-wide analyses of cortical Aβ PET status (+/-). Using data from participants in the Baltimore Longitudinal Study of Aging (BLSA; *n* = 196; age = 75.7 yrs. [SD = 8.3]) and the Atherosclerosis Risk in Communities study (ARIC; *n* = 272; age = 75.7 yrs. [SD = 5.2]) who had concurrent Aβ PET scans and plasma proteomic measurements on the SomaScan v4.0 (4,971 protein targets) and v4.1 (7,268 protein targets) platforms, respectively, we applied logistic regression models adjusted for demographic, physiological, and comorbid variables (Fig. 2a; sTable 1, 2; sFigure 1a). No proteins survived false discovery rate (FDR) correction for multiple comparisons, but seven displayed nominally significant (*p* < 0.05) associations across both cohorts, including four with directionally consistent relationships (Fig. 2b; sTables 3, 4). Unlike other plasma proteins linked to Aβ PET status, which display low or undetectable expression in CNS tissue and cell types, OMG is preferentially expressed by oligodendrocytes and neurons in white matter and the cerebral cortex (Fig. 2c; sTable 5; sFigure 1b). Meta-analyzed results indicated > 40% lower odds of Aβ positivity (+) per each standard deviation increase in this protein’s plasma abundance (OR = 0.56, 95% CI: 0.39, 0.81; Fig. 2b; sTable 6). Consistent with these results, an inverse relationship between CSF OMG (measured with the Olink platform) and CSF Aβ status (defined with Aβ_42/40_) was reported previously in the BioFINDER-2 cohort (*n* = 877) and the Johns Hopkins AD Research Center (*n* = 86) [14, 15].

After confirming that external studies have validated OMG measurements on the SomaScan platform (sTable 7), we leveraged publicly available data to demonstrate that OMG was lower at the RNA and protein level in AD-patient and -mouse brain tissue using bulk transcriptomics, mass spectrometry proteomics, and single-cell RNA-seq, where we found patterns of differential expression were most prominent in oligodendrocytes and neurons (Fig. 2d, e; sTables 8–10; sFigure 1c) [16–23]. In addition, elevated plasma OMG was associated with higher CSF Aβ_42_ (indicative of lower cortical Aβ), lower CSF total tau, and lower CSF pTau-181 among Emory AD Research Center participants (Emory-ADRC; *n* = 36, age = 66.5 yrs. [SD = 10.5]; Fig. 2f; sTables 2, 11), and with lower Braak stages – but not CERAD scores – in BLSA participants who underwent autopsy (*n* = 40; Fig. 2g; sTables 2, 12). Among participants with SomaScan and ADRD plasma biomarker data in the BLSA (Aβ_42/40_, NfL, GFAP *n* = 1,017; pTau181 *n* = 690) and ARIC (*n* = 1,299), plasma OMG showed consistent associations with higher GFAP, an indicator of reactive astrogliosis (Fig. 2h; sTables 2, 13). In the full BLSA proteomic sample (*n* = 1,275), we also detected robust relationships between OMG and several other plasma proteins implicated in ADRDs, including complexins (CPLX1, CPLX2), neuronal pentraxins (NPTXR1, NPTXR2), and TREM2 (Fig. 2i; sTables 2, 14). OMG is a component of outer myelin sheaths whose age-related decreases in the BLSA were dependent on comorbidities (Fig. 2j; sTable 15; sFigure 1d). In addition to physical interactions with other mediators of neurodegeneration (e.g., tau, reticulons), OMG can regulate neurite growth and be released from its glycosylphosphatidylinositol (GPI)-anchor as a secreted protein, suggesting its higher circulating abundance in the absence of AD pathology may be an indicator of preserved axonal structural integrity (Fig. 2k; sTable 16) [24–26].

### Plasma OMG is linked to lower odds of dementia

**Fig. 3:**
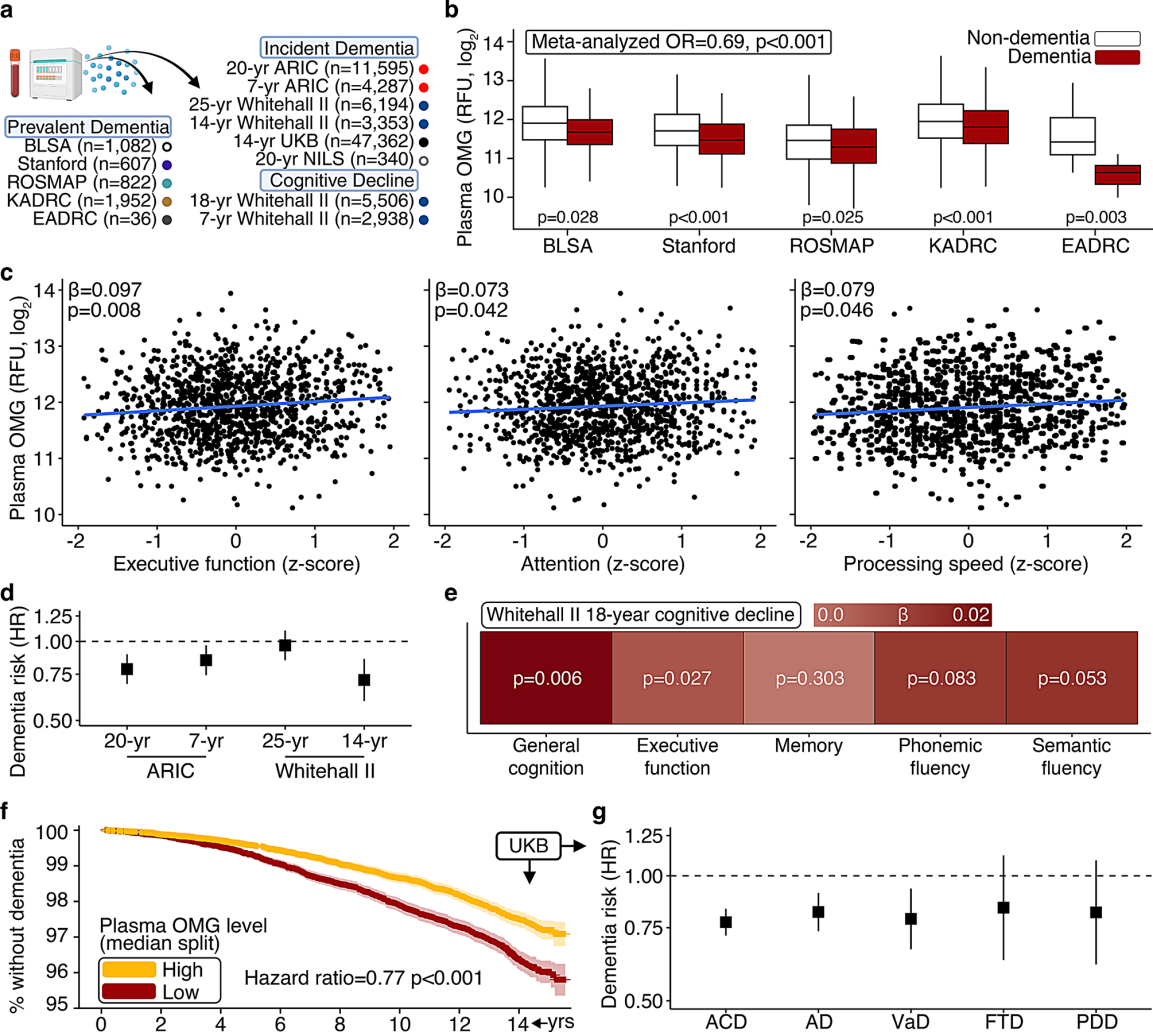
Plasma OMG is associated with lower odds of dementia, decreased risk of cognitive decline and dementia. **a**. Overview of cognition analyses. **b**. OMG plasma protein levels across dementia and non-dementia participants in the BLSA (*n* = 1,082), Stanford cohort (*n* = 607), ROSMAP (*n* = 822), Knight-ADRC (*n* = 1,952), and Emory-ADRC (*n* = 36). BLSA results derived from logistic regression adjusting for age, sex, race, education, *APOE*ε4, and a comorbidity index; ROSMAP, Knight-ADRC, Stanford, Emory-ADRC, and ADNI results derived from logistic regression adjusting for age, sex, and *APOE*ε4. Box plots: median, 25–75th quartiles; whiskers: 1.5× the IQR. **c**. Cognitive domain scores associated with plasma OMG in the BLSA (*n* = 1,275). Results derived from linear regression adjusting for age, sex, race, education, *APOE*ε4, and a comorbidity index. **d**. All-cause dementia risk associated with plasma OMG in ARIC (20-year *n* = 11,595; 7-year *n* = 4,287) and Whitehall II (25-year *n* = 6,194; 14-year *n* = 3,353). ARIC results derived from Cox regression adjusting for age, sex, race-center, education, *APOE*ε4, eGFR, and cardiovascular risk factors. Whitehall II results derived from Cox regression adjusting for age, sex, and ethnicity. Results are presented as hazard ratios (HRs) and 95% confidence intervals (CIs). **e**. Rates of cognitive decline associated with plasma OMG in Whitehall II (18-year *n* = 5,506). Results derived from linear mixed effects regression adjusting for age, sex, and ethnicity. **f**. All-cause dementia free risk associated plasma OMG in the UKB (14-year *n* = 47,362). Results are displayed according high/low OMG (median split); statistical analysis (and corresponding hazard ratio) employed OMG as a continuous variable. Results derived from Cox regression adjusting for age, sex, education, study site, *APOE*ε4, eGFR, and cardiovascular risk factors. **g**. Etiology-specific dementia risk associated with plasma OMG in the UKB (14-year *n* = 47,362). Results derived from Cox regression adjusting for age, sex, education, study site, *APOE*ε4, eGFR, and cardiovascular risk factors. Results are presented as HRs and 95% CIs. Reproduced by kind permission of UKB. All-cause dementia, ACD; Alzheimer’s disease, AD; FTD, frontotemporal dementia; hazard ratio, HR; PDD, Parkinson’s disease dementia; relative florescence units, RFU; VaD, vascular dementia

Given its enriched expression in the CNS and consistent associations with lower AD neuropathology, we asked if plasma OMG was related to prevalent dementia among participants with SomaScan proteomic data in the BLSA (*n* = 1,082, age = 66.4 yrs. [SD = 14.6]), the Stanford cohort (*n* = 607, age = 72.1 yrs. [SD = 8.1]), the Religious Orders Study/Rush Memory and Aging Project (ROSMAP; *n* = 822, age = 86.0 yrs. [SD = 6.4]), the Knight AD Research Center (Knight-ADRC; *n* = 1,952, age = 74.7 yrs. [SD = 9.9]), and the Emory-ADRC (*n* = 36; Fig. 3a; sTable 2). OMG was associated with a lower odds of dementia in each cohort using logistic regression models adjusted for demographic and physiological variables. Meta-analyzed results indicated that each standard deviation increase in plasma OMG abundance was associated with > 30% lower odds of dementia (OR = 0.68, 95% CI: 0.56, 0.80; Fig. 3b; sTable 17). These findings were supported by cognitive domain-specific analyses among BLSA participants, where higher plasma OMG was associated with better executive functioning, attention, and processing speed (Fig. 3c; sTable 18). As part of a cross-platform validation using Olink proteomic data (OMG Spearman’s rho = 0.88, *p* < 0.001 across platforms), we also found plasma OMG was associated with lower odds of dementia in the Emory-ADRC and a Hong Kong AD cohort (HKADC; *n* = 180; sTable 17) [7, 8]. These findings on the Olink platform align with our previous Olink results from the Women’s Health Initiative (WHI; *n* = 1,610), which reported higher plasma OMG abundance in cognitively resilient individuals at risk for AD (i.e., 80+ year-old *APOE4* carriers without cognitive impairment), and results from the UK Biobank (UKB; *n* = 46,218), which reported plasma OMG’s associations with lower odds of dementia, stroke, cerebrovascular disease, neurodegenerative conditions (including MS), and higher fluid intelligence (semantic, numeric; sTable 19) [8, 9]. CSF OMG abundance was not related to dementia status using SomaScan data from the AD Neuroimaging Initiative (ADNI; *n* = 655), the Stanford cohort (*n* = 438), or the Emory-ADRC (*n* = 36); however, CSF abundance of OMG in the Knight-ADRC (SomaScan; *n* = 752) and the Alzheimer Center Amsterdam cohort (mass spectrometry; *n* = 609) were related to higher and lower odds of dementia, respectively (sTables 2, 17) [8, 27]. These results derived from eight independent cohorts (BLSA, Stanford, ROSMAP, Knight-ADRC, Emory-ADRC, HKADC, WHI, UKB) indicate that individuals without dementia maintain higher plasma levels of OMG, while this protein’s predictive validity may be superior in plasma relative to CSF, consistent with some other brain-specific proteins (e.g., GFAP) [28].

### Plasma OMG is associated with decreased risk of cognitive decline and dementia

To determine plasma OMG’s capacity for identifying individuals at risk for future cognitive impairment, we examined its relationship with incident dementia risk among ARIC participants who had SomaScan plasma proteomic data collected at baseline and were followed over 20 years beginning in mid-life (*n* = 11,595) and over 7 years beginning in late-life (*n* = 4,287) (sTable 2). Using Cox proportional hazards models adjusted for demographic, physiological, and comorbid variables, we found OMG abundance in mid-life (age = 57.1 [SD = 5.7]) was related to decreased dementia risk over the subsequent two decades (hazard ratio [HR] = 0.78, 95% CI 0.69, 0.89), while OMG levels in late-life (age = 75.2 [SD = 5.0]) were similarly related to decreased dementia risk (HR = 0.85, 95% CI 0.74, 0.97; Fig. 3d; sTable 20; sFigure 2a). In stratified ARIC analyses, we found OMG’s associations with lower dementia risk across mid- and late-life were more robust among *APOE*4 non-carriers, Black participants, non-obese participants, female participants, individuals with one or more cardiovascular diseases, and individuals with higher levels of education (sTable 20; sFigure 2b). In a subset of participants with plasma measures of canonical ADRD biomarkers (*n* = 1,299), OMG’s association with 7-year dementia risk was not meaningfully affected by adjustment for Aβ_42/40_, pTau-181, or NfL, suggesting that its link to dementia is independent of Aβ deposition, tau phosphorylation, and axonal injury, respectively; however, the incorporation of GFAP as a covariate in statistical models enhanced OMG’s associations with late-life dementia risk by more than two-fold (sTables 2, 20). To further investigate the generalizability of OMG’s associations across races/ethnicities, we leveraged SomaScan plasma proteomic data from the National Institute for Longevity Sciences-Longitudinal Study of Aging (NILS-LSA) in Japan (*n* = 340; age = 66.7 [SD = 5.2]), where we did not detect OMG-dependent dementia risk over a 20 year follow up period (HR = 1.00, 95% CI 0.81, 1.24; sTables 2, 20).

Among participants with plasma SomaScan measurements in the Whitehall II cohort, we examined OMG’s relationship with etiology-specific dementia risk over 25 years beginning in mid-life (*n* = 6,194; age = 55.7 [SD = 6.0]) and over 14 years beginning in late-life (*n* = 3,353; age = 65.9 [SD = 6.0]). OMG levels were not associated with all-cause dementia (ACD) risk in mid-life (HR = 0.96, 95% CI 0.84, 1.10), but were linked to decreased ACD risk in late-life (HR = 0.78, 95% CI 0.67, 0.91; Fig. 3d; sTables 2, 19; sFigure 2c). Although OMG’s associations with AD (*p* = 0.078) and vascular (VaD; *p* = 0.127) dementias in late-life were not statistically significant, we observed a similar reduction in 14-yr dementia risk regardless of etiology: 18–22% lower risk with each standard deviation increase in plasma OMG. Leveraging additional data from Whitehall II, along with covariate-adjusted linear mixed effects models, we found higher plasma OMG predicted slower rates of cognitive decline over an 18 year follow up period in mid-life, especially in the domain of executive function (*n* = 5,506; age = 55.4 [SD = 5.9]; Fig. 3e; sTables 2, 20).

As part of a cross-platform validation to further investigate OMG’s association with etiology-specific dementia risk, we leveraged Olink proteomic data from the UKB (*n* = 47,362; age = 56.8 [SD = 8.2]), where we found that higher plasma OMG abundance was related to decreased risk of ACD, AD, and VaD over a 14-year follow up period (Fig. 3f; sTables 2, 20). OMG was associated with a similar reduction in 14-yr dementia risk regardless of etiology: 18–23% lower risk with each standard deviation increase in plasma OMG (Fig. 3g; sTable 20; sFigure 2d). OMG was not significantly associated with differential risk of Parkinson’s disease dementia (PDD; which can be considered a progression of Parkinson’s disease [PD]) or frontotemporal dementia (FTD), although effect sizes were similar to that of ACD, AD, and VaD. Additionally, we found higher OMG was linked to decreased risk of PD and any Parkinsonian condition (e.g., Multiple System Atrophy), but not Amyotrophic Lateral Sclerosis (ALS). Using data from three large, prospective cohort studies (ARIC, Whitehall II, UKB), these results indicate that participants with higher plasma OMG abundance display slower rates of cognitive decline and reduced risk of dementia up to two decades before symptom onset.

### Plasma OMG is related to preserved brain structure

**Fig. 4:**
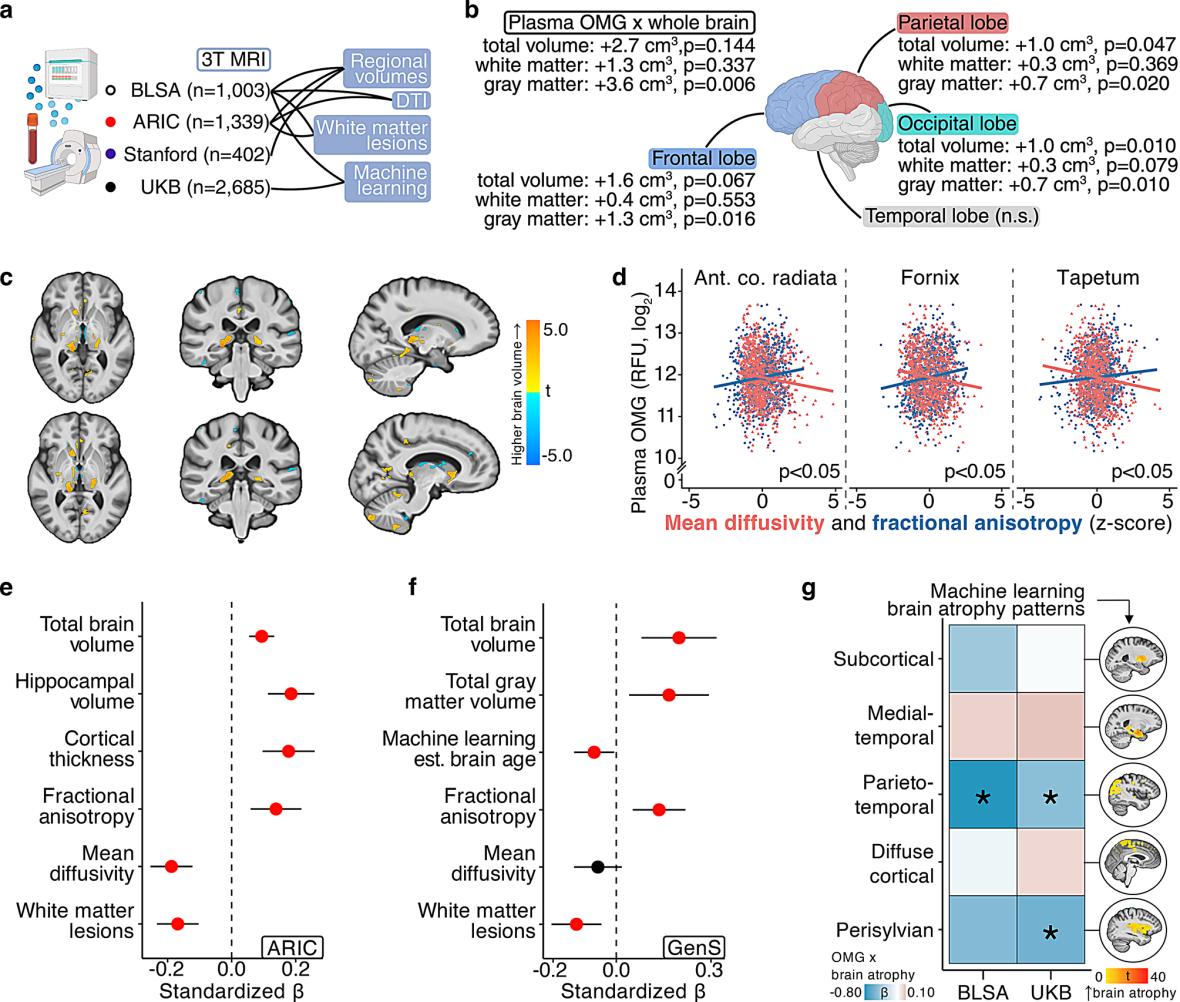
Plasma OMG is an indicator of preserved brain structure. **a**. Overview of 3T MRI analyses. **b**. Whole brain, lobar, and **c** voxel-wise brain volumes associated with plasma OMG in the BLSA (*n* = 1,003). Results derived from linear regression adjusting for age, sex, race, education, *APOE*ε4, a comorbidity index, and intracranial volume. **d**. White matter microstructure associated with plasma OMG in the BLSA (*n* = 1,003). Results derived from linear regression adjusting for age, sex, race, education, *APOE*ε4, and a comorbidity index. **e**. Structural brain measurements associated with plasma OMG in the ARIC (*n* = 1,339). Results derived from linear regression adjusted for age, sex, race-center, education, *APOE*ε4, eGFR, intracranial volume, and cardiovascular risk factors. Results are presented as standardized beta coefficients (β) and 95% confidence intervals (CIs). **f**. Structural brain measurements associated with plasma OMG in GenS (*n* = 1,065). Results derived from linear mixed-effects regression adjusting for kinship matrix, age, sex, depression diagnosis, clinic study site, sample storage time, intracranial volume, and processing artifacts. Results are presented as standardized βs and 95% CIs. **g**. Machine learning-derived neuroimaging signatures of brain atrophy associated with plasma OMG in the BLSA (*n* = 1,003) and the UKB (*n* = 2,685). BLSA results derived from linear regression adjusting for age, sex, race, education, *APOE*ε4, and a comorbidity index. UKB results derived from linear regression adjusting for age, sex, total household income, *APOE*ε4, eGFR, and cardiovascular risk factors. *indicates *p* < 0.05

To better understand the neurobiological basis for OMG’s consistent links with cognition and dementia risk, we characterized the associations of plasma OMG with structural neuroimaging obtained with 3T MRI (Fig. 4a). Using BLSA SomaScan data (*n* = 1,003; age = 65.7 [SD = 14.9]) and covariate-adjusted linear regression models, we found plasma OMG abundance was related to higher regional brain volumes, especially gray matter in frontal, parietal, and occipital lobes, with voxel-based analyses showing OMG was also strongly associated with higher volume in the precuneus (i.e., where Aβ accumulation preferentially starts) and certain subcortical structures (e.g., thalamus, caudate; Fig. 4b, c; sTables 2, 21; sFigure 4, 4) [29]. Plasma OMG levels were also associated with lower white matter hyperintensity volume, and diffusion tensor imaging revealed this plasma protein’s relationship with preserved white matter microstructure (i.e., higher fractional anisotropy, lower mean diffusivity), especially in the anterior corona radiata, fornix, and tapetum (Fig. 4d; sTable 21).

We observed similar OMG-related patterns using ARIC SomaScan data (*n* = 1,339; age = 76.1 [SD = 5.2]), namely higher regional brain volumes, greater cortical thickness, and preserved white matter microstructure (Fig. 4e; sTables 2, 22). Previous ARIC results reported plasma OMG’s associations with lower white matter hyperintensity volume, microhemorrhages, and infarcts, as well as lower white matter hyperintensity volume when OMG is measured 18 years prior to MRI scan; similarly, plasma OMG in the Cardiovascular Health Study (SomaScan; *n* = 765) has been linked to lower white matter lesions at baseline and slower increases in white matter lesions over 5 years (sTables 22) [10]. Using computed results from the Generation Scotland (GenS) cohort (*n* = 1,065; age = 59.9 [SD = 9.6]), where participants had SomaScan and 3T MRI data collected concurrently, we found plasma OMG was associated with higher total brain volume, gray matter volume, and fractional anisotropy, as well as lower machine learning-estimated brain age and white matter hyperintensity volume (Fig. 4f; sTables 23) [11].

As part of a cross-platform validation to investigate plasma OMG’s associations with structurally distinct and clinically relevant patterns of neurodegeneration, we calculated five machine learning-derived neuroimaging signatures in the BLSA and the UKB (*n* = 2,685; age = 63.7 [SD = 7.7]). These measures capture the co-expression of multi-dimensional atrophy patterns that predict age-related clinical traits and neurodegenerative disease risk in large cohort studies, including clinical progression and dementia diagnosis [30]. Plasma OMG levels in the BLSA (SomaScan) and UKB (Olink) were associated with lower parieto-temporal atrophy, while OMG in UKB was additionally related to lower perisylvian atrophy (Fig. 4g; sTables 2, 24). Prior results in the UKB also reported plasma OMG’s associations with lower white matter hyperintensity volume [12]. To compare the strength of OMG’s associations with structural neuroimaging when measured across different biofluids, we used data from a subset of Stanford participants who had 3T MRI and plasma (*n* = 402) or CSF (*n* = 173) SomaScan data collected at the same study visit; we found statistically insignificant but stronger relationships with brain structure when OMG was measured in CSF compared to plasma (sTable 25). As evidenced by complementary structural neuroimaging modalities across five independent cohorts (BLSA, ARIC, CHS, GenS, UKB), these findings indicate that individuals with higher plasma levels of OMG exhibit lower levels of neurodegeneration and preserved white matter integrity.

### Plasma OMG is associated with lower odds of multiple sclerosis

Given evidence for the role of myelin-specific proteins in autoantibody-mediated neuroinflammatory conditions, along with its reported associations with lower odds and risk of MS in the UKB (Olink), we examined whether plasma OMG was associated with prevalent MS, structural neuroimaging, and neurological task performance among participants with SomaScan proteomic data from the Johns Hopkins Multiple Sclerosis Center (JHMSC; *n* = 397; age = 51.6 [SD = 10.8]; sTable 2) [8, 31–33]. In covariate-adjusted logistic regression models, plasma OMG was associated with lower odds of MS (MS: OR = 0.42, 95% CI 0.25, 0.71, *p* = 0.001), a finding that was slightly more evident with progressive MS (OR = 0.37, 95% CI 0.20, 0.71, *p* = 0.003) than with relapse-remitting MS (OR = 0.44, 95% CI 0.25, 0.78, *p* = 0.005; sTable 26; sFigure 5a). Among individuals with MS, higher plasma OMG abundance was related to higher thalamic brain volume, and showed non-significant trends with higher subcortical gray matter volume (*p* = 0.088) and lower white matter lesion volume (*p* = 0.098). Elevated plasma OMG was also related to faster performance on tests of manual dexterity and walking speed, and showed trending associations with higher processing speed (*p* = 0.079; sFigure 5b).

### OMG protein abundance across biofluids

Using plasma and CSF SomaScan data collected from the same participants at the same study visit in the Johns Hopkins Neurology cohort (JHN; *n* = 194), the Knight-ADRC (*n* = 268), and the Stanford cohort (*n* = 212), we found OMG was higher in CSF compared to plasma and weakly correlated across biofluids (Spearman’s rho 0.07–0.17; sTable 2; sTable 27). Consistent with our previous observations of plasma OMG in the BLSA (Fig. 2j; sFigure 1d), OMG displayed an inverse relationship with age; however, the statistical significance of this decrease tended to be more robust in plasma compared to CSF (sTable 27). These results suggest that OMG is expressed in the CNS and migrates into peripheral circulation where its measurement is more sensitive to broader reductions in OMG protein levels, which could explain its superior predictive validity in plasma relative to CSF (sTable 17).

### OMG’s CSF proteo-biological signature

**Fig. 5:**
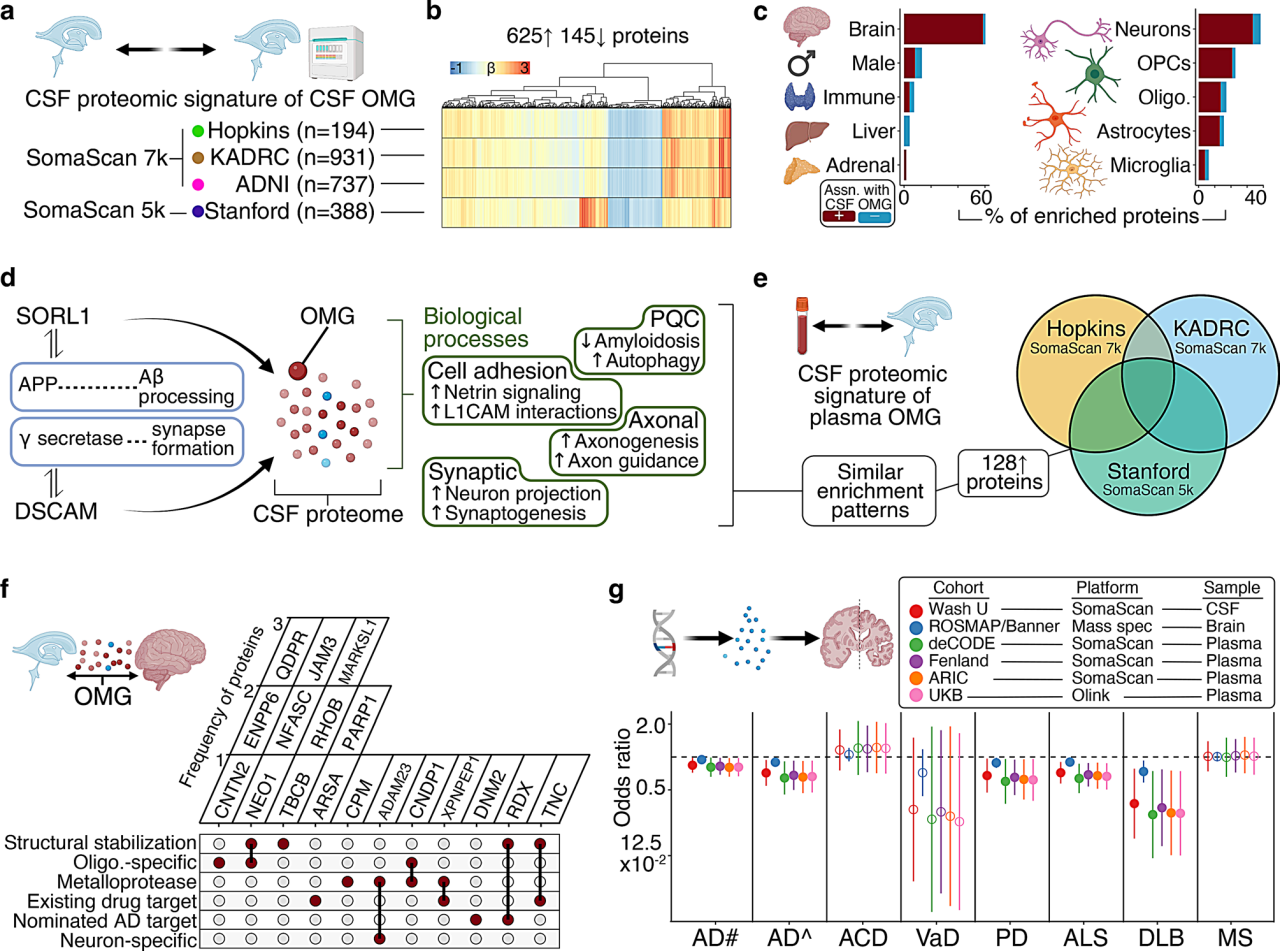
OMG’s CSF and brain proteomic signatures, and causal genetic evidence. **a**. Overview of CSF analyses. **b**. The CSF proteomic signature (FDR *p* < 0.05) of CSF OMG in JHN (*n* = 194), Knight-ADRC (*n* = 931), ADNI (*n* = 737), and Stanford (*n* = 388). Results derived from linear regression adjusting for age and sex. Dendrogram reflects hierarchical clustering using Euclidean distances. **c**. Tissue- and cell-specific enrichment patterns of OMG’s CSF proteomic signature. **d**. Upstream regulators and enriched biological processes of OMG’s CSF proteomic signature. Results derived from publicly available databases (e.g., gene ontology) and Ingenuity Pathway Analysis. **e**. The CSF proteomic signature (FDR *p* < 0.05) of plasma OMG in JHN (*n* = 194), Knight-ADRC (*n* = 268), and Stanford (*n* = 212). Results derived from linear regression adjusting for age and sex. **f**. Structural stabilization and metalloprotease proteins of OMG’s conserved brain tissue proteomic signature in the BLSA (*n* = 40), as well as information related to their cell-specificity, whether they are targets of existing drugs, and whether they are nominated therapeutic targets for AD. Results derived from linear regression adjusting for age and most recent cognitive performance, and from the Accelerating Medicines Partnership Program for AD. **g**. Two-sample MR established the causal relationships of cis-regulated OMG abundance and neurodegenerative diseases. Results derived from Wald ratio estimates. pQTLs were obtained from previously reported GWAS summary statistics of proteomic measurements that were expressed on log_10_ (WashU, CSF), log_2_ (ROSMAP/Banner, brain tissue; ARIC, plasma; UKB, plasma), or rank-based inverse normal transformation (deCODE, plasma; Fenland, plasma) scales. AD# reflects summary statistics from Bellenguez et al., 2022, and AD^ reflects summary statistics from Kunkle et al., 2019. Results are presented as odds ratios (ORs) and 95% confidence intervals (CIs)

Given its brain-specific expression patterns, we hypothesized that OMG’s mechanistic relevance to neurodegeneration is primarily mediated in the CNS rather than in peripheral circulation. Therefore, we focused on investigating OMG’s CSF proteomic signature to identify biological processes that might account for its relationships with neurocognitive outcomes (Fig. 5a). Using CSF SomaScan v4.1 data (7,039 protein targets) from JHN (*n* = 194), the Knight-ADRC (*n* = 931), and ADNI (*n* = 737), along with age and sex adjusted linear regression models, we identified 1,646 CSF proteins that showed consistent directional associations with CSF OMG levels across these three cohorts (FDR < 0.05). To obtain a more reliable CSF proteomic signature of OMG, we incorporated data from the Stanford cohort, where SomaScan v4.0 data were available. 1,161 of these proteins were measured on the SomaScan v4 platform (4,830 protein targets) in CSF of Stanford cohort participants (*n* = 388), and 770 showed consistent directional associations with CSF OMG (625 and 145 positive and negative associations, respectively; FDR < 0.05; Fig. 5b; sTable 28; sFigure 6a).

In the CSF proteomic signature of CSF OMG (770 proteins), we confirmed enrichment of brain-, neuronal-, and oligodendroglial-specific proteins relative to other organs and CNS cell types, respectively (Fig. 5c; sTable 29). Consistent with our previous observations of plasma OMG in the BLSA (Fig. 2i), we detected robust relationships between CSF OMG and several other CSF proteins implicated in ADRDs, including complexins (CPLX1, CPLX2), neuronal pentraxins (NPTXR1, NPTXR2), and TREM2 (sFigure 6b). OMG also displayed inverse relationships with CSF markers of blood-brain-barrier (BBB) breakdown, including albumin, immunoglobulin G, and fibrinogen. With publicly available databases and Ingenuity Pathway Analyses (IPA), we detected consistent upregulation of several biological processes including: axonal (e.g., axon guidance) and synaptic (e.g., synaptogenesis) integrity, cell adhesion (e.g., L1CAM signaling), and protein quality control (PQC; e.g., autophagy; sTable 30; sFigure 7a, b). Results from IPAs causal network analyses revealed OMG’s CSF proteomic signature and its enriched biological processes were most dependent on upstream regulation by SORL1 (Sortilin Related Receptor 1), a CNS lipoprotein receptor whose loss of function AD-risk variants can impair endosomal trafficking of Aβ, and DSCAM (Down syndrome cell adhesion molecule), a trisomy 21-related neuronal cell adhesion molecule whose interactions with gamma secretase can modulate synapse formation (Fig. 5d; sTable 31) [34, 35].

The CSF proteomic signature of plasma OMG was also examined using similar age and sex adjusted linear regression models, along with plasma and CSF SomaScan data collected at the same study visit from JHN (*n* = 194), the Knight-ADRC (*n* = 268), and the Stanford cohort (*n* = 212; sTable 2). 128 CSF proteins showed consistent directional associations with plasma OMG abundance across these three cohorts (all positive associations; FDR < 0.05; Fig. 5e; sTable 32). Plasma OMG’s inverse relationships with CSF markers of BBB breakdown were attenuated compared to CSF OMG. This pattern, as well as the differential frequency of CSF proteins associated with CSF OMG abundance (770 proteins) relative to plasma OMG abundance (128 proteins), may be attributed to OMG’s weak correlations across biofluids (sTable 27). Despite limited overlap, we observed similar enrichment of tissues (i.e., brain), cells (i.e., neurons, oligodenodrocytes), pathways (i.e., axon guidance, synaptogenesis) and upstream regulators (i.e., SORL1, DSCAM) in the CSF proteomic signature of plasma OMG (sTables 33–35). These results suggest that OMG abundance is reflective of broader neuronal and oligodendroglial mechanisms which provide resiliency against neurodegeneration and its underlying disease processes (e.g., perturbations in amyloid processing, compromised neuronal structural integrity) by promoting axonal stability, synaptic functioning, intercellular adhesion, and proteostasis.

### OMG’s conserved proteomic signature in brain tissue

As part of a cross-biospecimen and cross-platform validation of OMG-related neurobiological processes, we applied age and sex adjusted linear regression models to determine whether CSF proteins linked to CSF OMG levels (SomaScan) were also associated with brain tissue OMG levels in BLSA participants who underwent autopsy (mass spectrometry; *n* = 40; sTable 2). Unlike preceding OMG proteomic signatures in CSF, which primarily captured soluble protein measurements, these mass spectrometry data were derived from the detergent insoluble sample fraction. Of the 770 CSF proteins associated with CSF OMG abundance in preceding analyses, 388 were quantified in brain tissue, but only 25 (6%) showed consistent directional associations with OMG (23 positive and 2 negative associations; FDR < 0.05; sTable 36; sFigure 8a), suggesting that the proteomic correlates of OMG are sensitive to assay type, sample preparation (i.e., soluble/insoluble), and/or vary across the parenchymal and CSF milieu. Despite limited overlap and orthogonal proteomic techniques, in OMG’s conserved brain tissue proteomic signature (25 proteins), we observed similar patterns of tissue-, cell-, and pathway-enrichment (e.g., axon guidance), along with additional enrichment of several immune processes (e.g., granulocyte adhesion; sTables 37–39; sFigure 8b). OMG’s conserved brain tissue proteomic signature was most strongly and positively associated with mediators of structural stabilization (e.g., JAM3) and metalloproteases (e.g., ADAM23), several of which were also oligodendrocyte-specific, targets of existing drugs, and/or nominated AD therapeutic targets (Fig. 5f; sTable 40; sFigure 8c). These findings further suggest that OMG abundance is reflective of broader neurobiological mechanisms which afford resiliency against neurodegeneration by preserving neuronal structural integrity, especially axonal projections.

### Genetic evidence of OMG’s causal role in neurodegeneration

Having identified biological processes that might account for OMG’s neuroprotective relationships, we then examined if genetic variation that influences OMG protein abundance plays a causal role in neurodegenerative diseases. Due to our working hypothesis that OMG’s mechanistic relevance is more likely mediated in the CNS rather than in peripheral circulation, we prioritized investigation of cis variants in or near the *OMG* gene that influence CSF OMG levels, but we also investigated cis regulators of brain and plasma OMG levels. 15 OMG protein quantitative trait loci (pQTLs) were identified in a GWAS of CSF SomaScan levels led by Washington University in St. Louis (WashU; *n* = 3,506) [36]. CSF pQTLs showed directionally consistent associations with dorsolateral prefrontal cortex OMG levels in a ROSMAP/Banner cohort (mass spectrometry; *n* = 716), as well as plasma OMG levels in the deCODE cohort (SomaScan; *n* = 35,559), the Fenland cohort (SomaScan; *n* = 10,708), ARIC (SomaScan; *n* = 7,597), and the UKB (Olink; *n* = 54,219; sTable 41; sFigure 9) [37–41]. These SNPs have been associated with cognitive function and neurodegenerative conditions across multiple GWAS (sTable 42). Consistent with OMG’s neuroprotective associations in preceding analyses, several cis OMG variants (rs4263003, rs7212264, rs4795587) linked to lower protein levels in CSF, brain tissue, and plasma were also associated with a higher odds of dementia among BLSA participants with available genotyping data (*p* < 0.05; *n* = 873; sTable 2, 43; sFigure 9).

To formally test the causal role of CSF OMG abundance, we conducted two-sample Mendelian randomization (MR) using GWAS summary statistics of AD (one from 2022 and another from 2019), ACD, VaD, PD, ALS, DLB, and MS. 1 cis pQTL (rs72813607) was retained after pruning variants in linkage disequilibrium (LD; *r*^2^ < 0.05); because this variant was not annotated in DLB summary statistics, another pQTL in LD was selected (*r*^2^ = 1.0; rs72813627). Both of these variants have been linked to lower cortical OMG RNA levels in the Genotype-Tissue Expression project (GTEx; *n* = 268) and higher odds of demyelinating diseases in the UKB (*n* = 395,464; sTable 42; sFigure 10) [37]. We found evidence supporting a causal relationship of higher cis-regulated CSF OMG abundance with lower odds of AD (2022 AD GWAS: OR = 0.84, 95% CI: 0.72, 0.98; 2019 AD GWAS: OR = 0.72, 95% CI: 0.54, 0.94), PD (OR = 0.68, 95% CI: 0.47, 0.97), ALS (OR = 0.72, 95% CI: 0.57, 0.90), and DLB (OR = 0.37, 95% CI: 0.18, 0.78), but not ACD (OR = 1.16, 95% CI: 0.75, 1.78), VaD (OR = 0.33, 95% CI: 0.07, 1.50), or MS (OR = 1.01, 95% CI: 0.74, 1.39; Fig. 5g; sTable 44). To assess the causal role of brain OMG abundance, we repeated MR using cis pQTLs from a ROSMAP/Banner cohort. After pruning, the single retained cis pQTL (rs72815624) was in in LD (*r*^2^ = 1.0) with the pQTL used for CSF MR; this brain tissue pQTL showed similar associations with lower cortical OMG RNA levels and higher odds of demyelinating diseases (sTable 42; sFigure 10). We found consistent evidence supporting a causal relationship of higher cis-regulated brain OMG abundance with lower odds of AD (2022 AD GWAS: OR = 0.95, 95% CI: 0.90, 0.99; 2019 AD GWAS: OR = 0.89, 95% CI: 0.82, 0.98), PD (OR = 0.88, 95% CI: 0.79, 0.98), ALS (OR = 0.90, 95% CI: 0.84, 0.96), and DLB (OR = 0.74, 95% CI: 0.58, 0.93), but not ACD (OR = 1.05, 95% CI: 0.91, 1.21), VaD (OR = 0.72, 95% CI: 0.44, 1.18), or MS (OR = 1.00, 95% CI: 0.91, 1.11; Fig. 5g; sTable 44).

To assess the causal role of plasma OMG abundance, we repeated MR again using cis pQTLs from deCODE, Fenland, ARIC, and the UKB. After pruning, the single cis pQTL retained from deCODE (rs12449302), Fenland (rs72815624), and ARIC (rs17884466) were in LD (*r*^2^ = 1.0) with the pQTL used for CSF MR, while the variant retained from the UKB was the same pQTL used for CSF MR (rs72813607). These variants have been associated with lower cortical OMG RNA levels and neurocognitive phenotypes in multiple GWAS (sTable 42; sFigure 10). We found evidence supporting a causal relationship of higher cis-regulated plasma OMG abundance with lower odds of AD, PD, ALS, and DLB using each pQTL dataset. In meta-analyzed results, we found evidence supporting a causal relationship of higher cis-regulated plasma OMG abundance with lower odds of AD (2022 AD GWAS: OR = 0.82, 95% CI: 0.75, 0.89; 2019 AD GWAS: OR = 0.67, 95% CI: 0.59, 0.78), PD (OR = 0.64, 95% CI: 0.53, 0.77), ALS (OR = 0.68, 95% CI: 0.61, 0.77), DLB (OR = 0.33, 95% CI: 0.23, 0.48), and VaD (OR = 0.29, 95% CI: 0.13, 0.65), but not ACD (OR = 1.19, 95% CI: 0.95, 1.49) or MS (OR = 1.02, 95% CI: 0.86, 1.20; Fig. 5g; sTable 44). Using six sets of genetic instruments (WashU, deCODE, Fenland, ARIC, UKB, ROSMAP/Banner) derived from different biofluids, biospecimens, and proteomic platforms (CSF, brain tissue, plasma; SomaScan, Olink, mass spectrometry), these results indicate that OMG abundance plays a causal, protective role in multiple neurodegenerative diseases.

## Discussion

**Fig. 6:**
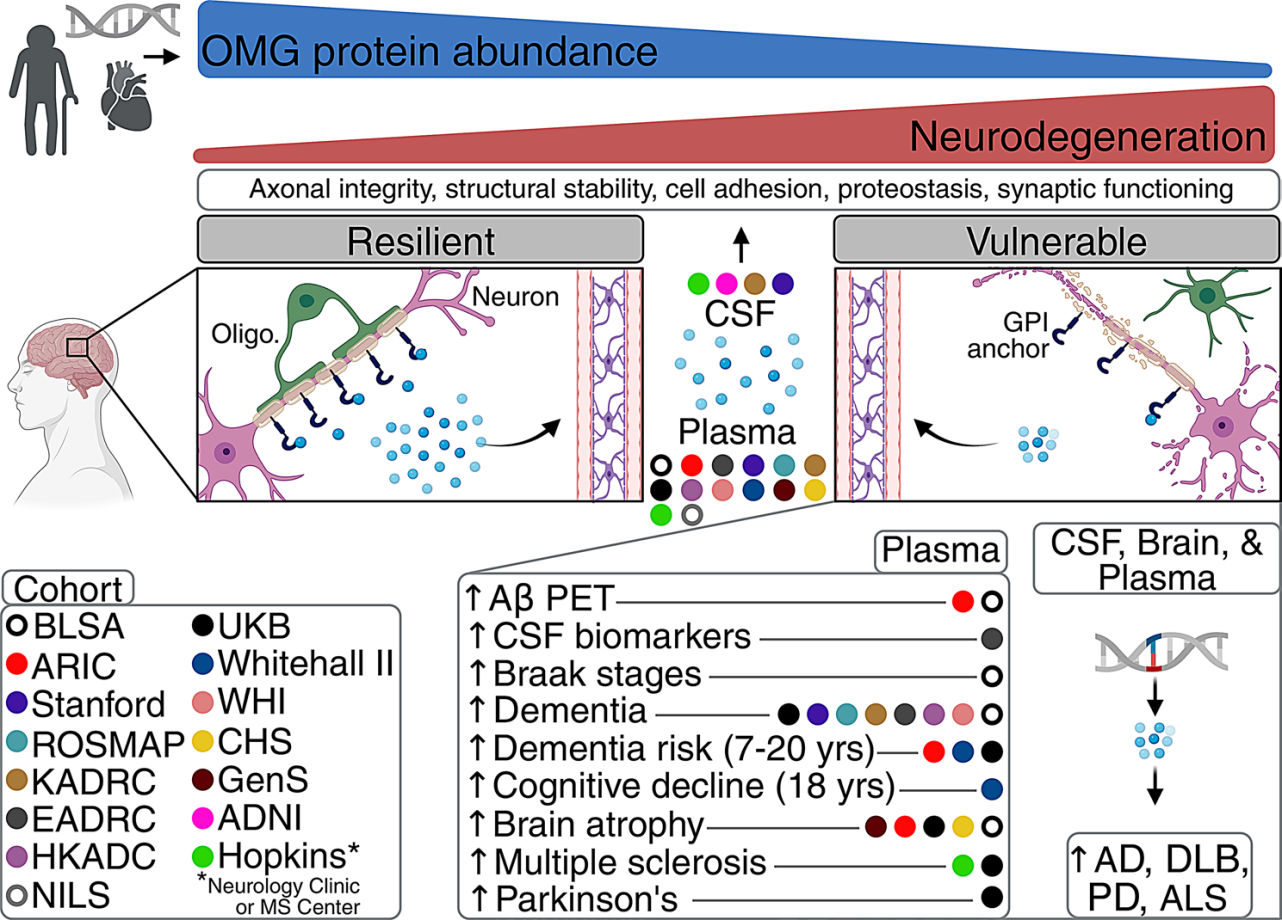
Working model of OMG. Lower abundance of the brain-specific OMG protein (which can be attributed to older age, cardiovascular comorbidities, and/or genetic regulation) is associated with compromised axonal integrity, along with impaired structural stability, cell adhesion, proteostasis, and synaptic functioning. As evidenced by complementary neurocognitive phenotypes across independent cohorts, this waning neurodegenerative resiliency is reliably captured by OMG abundance in peripheral circulation. Genetic variation in or near the *OMG* locus which represses its protein abundance across biofluids and brain tissue enhances vulnerability to neurodegenerative diseases. Alzheimer’s disease neuroimaging Initiative, ADNI; Atherosclerosis risk in Communities study, ARIC; Baltimore Longitudinal study of Aging, BLSA; cardiovascular Health study, CHS; Emory Alzheimer’s disease Research Center, EADRC; Generation Scotland study, GenS; Hong Kong Alzheimer’s disease cohort, HKADC; Knight Alzheimer’s disease Research Center, KADRC; Religious Orders study/Memory and Aging Project, ROSMAP; UK Biobank, UKB; WHI, Women’s Health Initiative

With high-throughput plasma proteomic data from over a dozen cohorts, we detected a brain-specific protein called OMG that was associated with lower cortical Aβ PET levels, and validated this protein’s relationship with lower odds of dementia, decreased dementia risk, slower cognitive decline, preserved brain structure, and lower odds of MS. OMG’s multi-cohort, CSF proteomic signature reflected broader neuroprotective mechanisms that were also observed in brain tissue, especially processes related to axonal structural integrity. Genetic methods supported the co-regulation of OMG across biofluids and brain tissue as causally protective against multiple neurodegenerative diseases (AD, PD, ALS, DLB). Together, these findings identify OMG as a mechanistically relevant determinant of neurodegenerative resiliency, which is reliably captured by its plasma abundance (Fig. 6).

The current investigation demonstrates how a single, reliable proteomic target can reveal insights into molecular mechanisms underlying the maintenance of neurocognitive functioning. Despite differences in diagnostic methodology between cohorts (e.g., clinical adjudication, electronic health records, etc.,), plasma OMG abundance was consistently linked to approximately 30% lower odds of dementia cross-sectionally and 20% decreased risk of dementia over 14–20 year follow up periods. The consistency of the OMG effect estimates despite the absence of a standardized diagnostic criteria across studies supports the reproducible nature of our findings. While the predominance of cross-sectional analyses limits direct causal inference, our longitudinal analyses of cognitive trajectories and incident dementia supports the mechanistic link between higher circulating levels of OMG and preserved cognitive functioning. Compared to existing AD plasma biomarkers that have been developed over limited follow-up times to specifically detect near-term (≤8 years) or prevalent AD (e.g., a standard deviation higher pTau-217 corresponds to 139% higher odds of Aβ+ PET in cognitively unimpaired BioFINDER-2 participants [*n* = 350]), OMG’s utility appears limited (i.e., a standard deviation higher OMG only corresponds to 40% lower odds of Aβ+ PET in cognitively unimpaired BLSA participants [*n* = 196]) [42, 43]. However, OMG’s ability to predict incident, etiology-agnostic dementia risk up to two decades before symptom onset could suggest its utility for the early detection of vulnerable populations who can benefit most from disease-modifying therapies [44]. Beyond dementia, OMG’s broader associations with complementary neurodegenerative phenotypes and MS indicates this protein’s potential as a non-specific biomarker of underlying axonal structural integrity in older adults, similar to NfL, but with additional mechanistic relevance. While we found plasma OMG levels predicted decreased risk of ACD, AD, VaD, PD and any Parkinsonian condition, its lack of statistically significant associations with PDD and FTD may have been attributed to small number of cases for these conditions, as suggested by the similar effect sizes we observed with each outcome. Given that many neurodegenerative conditions are characterized by extended prodromal periods [45, 46], this single protein’s capacity to track neurological outcomes over time suggests OMG could serve a marker for the health of large, myelinated axons, whose integrity can be compromised early in different disease subtypes [47].

Although its mechanistic relevance to neurodegeneration is likely mediated in the CNS due to its brain-specific expression patterns, we found that the preserved levels of OMG among cognitively resilient and younger participants were more apparent in plasma than in CSF, consistent with other brain-specific proteins like GFAP [28]. Controlling for plasma GFAP enhanced OMG’s detection of individuals at risk for dementia, suggesting that peripheral OMG abundance accounts for unique biological variance in dementia risk. This hypothesis is further supported by the absence of reactive astrocytic processes in OMG’s CSF, plasma, or brain proteomic signatures, despite robust, positive OMG-GFAP correlations in plasma. Consistent with its inverse relationships with cortical Aβ PET status, we found positive associations of plasma OMG with plasma Aβ_42/40_ (indicative of lower cortical Aβ) in ARIC; however, unexpected positive associations with plasma pTau-181 and NfL were also detected in BLSA. Although further investigation is needed, we speculate that these results may have occurred for a number of different reasons, including idiosyncrasies of the cohort or transient plasma OMG elevations in preclinical or early AD as part of a compensatory protective response. Given its broad neuroprotective associations and the observed positive correlations of CSF OMG with CSF markers of BBB integrity, this protein’s superior sensitivity in plasma is unlikely due to passive diffusion dynamics. Rather, the enhanced sensitivity of plasma OMG (relative to CSF OMG) may be due to differential clearance mechanisms across biofluids. Individuals resilient to neurodegeneration might not only have more OMG available for efflux from the CNS, but also preserved active transport processes that contribute to greater accumulation of peripheral OMG abundance relative to older adults who are vulnerable to neurodegeneration. OMG’s superior sensitivity in plasma may also be related to preanalytical factors, such as decreased degradation or enhanced stability of protein measurements in plasma compared to CSF. Given the accessibility and scalability of plasma- versus CSF-derived samples, future investigations are needed to determine why some CNS proteins like OMG and GFAP correlate better with neurological outcomes when measured systemically rather than centrally; in turn, this may further illuminate mechanistic mediators of neurodegenerative diseases, and enhance detection of cost-effective, noninvasive, and clinically relevant biomarkers.

Given its causal evidence derived from two sample MR, its CNS-specificity, and its inherent extracellular localization, targeting OMG may be a viable therapeutic strategy. After its reported inhibition of neurite outgrowth in vitro in 2002 (findings which were challenged by later in vivo studies), several academic and private teams initiated development of OMG antagonists with the hopes of inducing axon regeneration following spinal cord injury, but there are no currently approved drugs or therapies in clinical development that specifically target OMG [26, 48, 49]. As evidenced by recent clinical trials, glucagon-like peptide-1 (GLP-1) analog treatment can increase OMG levels, whereas dietary intervention alone cannot [50, 51]. Rather than simply delivering higher amounts of OMG protein to the CNS, boosting its canonical role in axonal integrity by increasing its retention or integration in myelin sheaths may be more likely to offer therapeutic benefits. This might be achieved with phospholipase C inhibitors that could inhibit the release of OMG from its GPI anchor, or with gene therapies that selectively induce endogenous expression of OMG in older age. Additional validation studies are required to further illustrate OMG’s capacitates as a non-specific biomarker and/or a causal moderator worthy of drug development.

While our study has notable strengths, including state-of-the art proteomic data across independent cohorts, the inclusion of multi-modal neurodegenerative phenotypes, the integration of plasma-CSF-brain proteomic signatures, and genetic techniques to support causality, our study also has several limitations. First, we lacked an experimental paradigm to validate the temporal precedence of changes in OMG protein levels. Despite the lack of commercially or privately available OMG therapeutics and mouse models, future in vitro and in vivo studies are needed to verify the consequences of manipulating OMG abundance on functional, disease-related outcomes (e.g., axonal integrity, electrophysiology, cognitive performance etc.,). Second, a liberal threshold of *p* < 0.05 was applied to our discovery analyses using Aβ PET in two community-based samples; we leveraged data from over a dozen cohorts to demonstrate the reliability of OMG’s relationships with neurocognitive outcomes, but its association with PET-defined amyloidosis did not survive correction for multiple comparisons. Third, because the cohorts used in our study were predominantly of European ancestry, our findings may not be as generalizable to individuals of non-European ancestries. Relatedly, while our triangulation approach leveraged data from different community-based and clinical cohorts from North America, Europe, and Asia, we were unable to completely control for study specific selection, inclusion, and survivor bias; regarding the latter, if OMG is also associated with lower mortality and/or better general health, the absence of the most unhealthy individuals with the lowest levels of OMG and greatest dementia risk may have biased associations toward the null. Despite these limitations, our results implicate OMG as a mechanistic determinant of neurodegenerative resiliency among older adults, which is reliably captured by its abundance in peripheral circulation.

## supplementary material

Below is the link to the electronic supplementary material.

Supplementary Material 1

Supplementary Material 2

Supplementary Material 3

Supplementary Material 4

Supplementary Material 5

Supplementary Material 6

Supplementary Material 7

Supplementary Material 8

Supplementary Material 9

Supplementary Material 10

Supplementary Material 11

Supplementary Material 12

## Data Availability

Data generated in the present investigation are included in this article, available on reasonable request, or in an online public repository. Study-specific data availability statements are available in the Supplementary Methods.

## Code availability

No original code was developed for this study, and no custom code or mathematical algorithm was central to its conclusions. Analyses used publicly available R packages, including stats (4.4), tidyverse (2.0), nlme (3.1), TwoSampleMR (0.6.6), and ggplot2 (3.5), with basic functions (e.g., lm, full_join, mr, geom_point etc.,).
