# Supplementary figures and images for: OMG! A proteomic determinant of neurodegenerative resiliency
Source: Mol Neurodegener. 2026 Jan 5;21:9. doi: 10.1186/s13024-025-00921-1 (PMC12870269; doi:10.1186/s13024-025-00921-1)

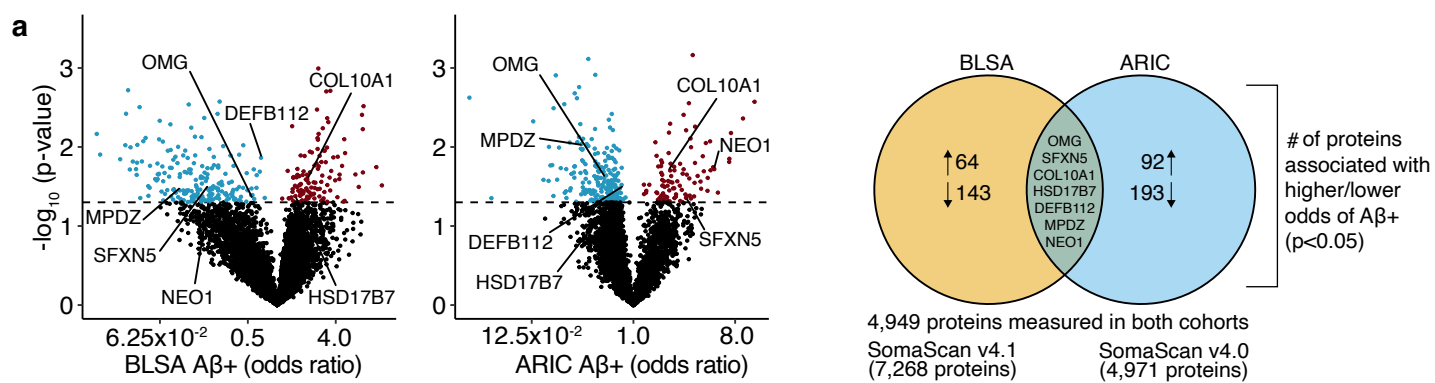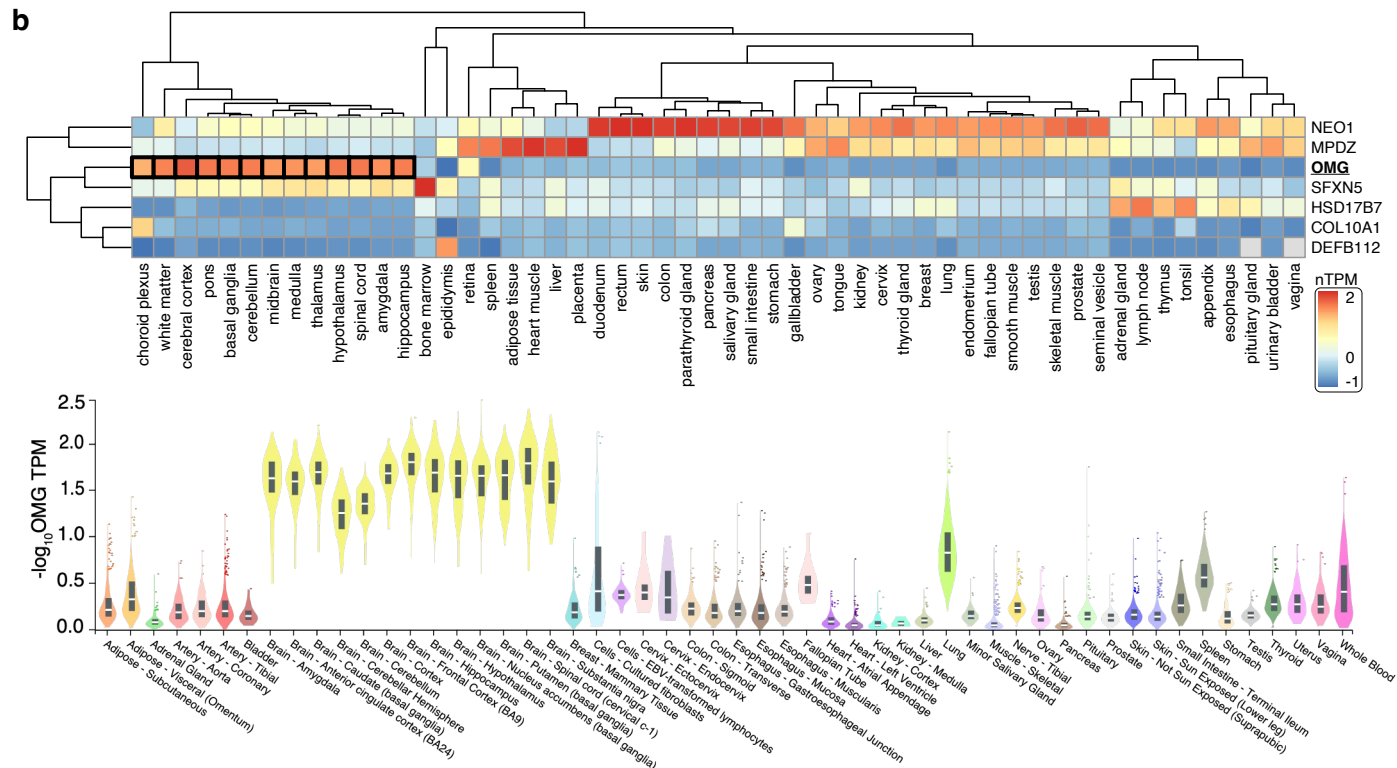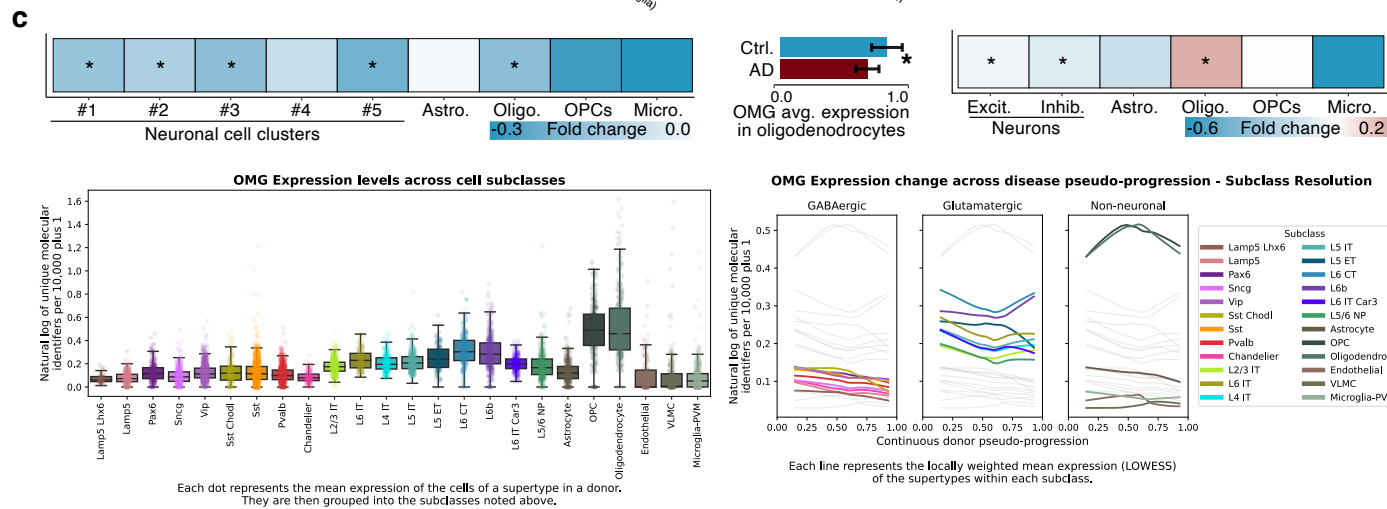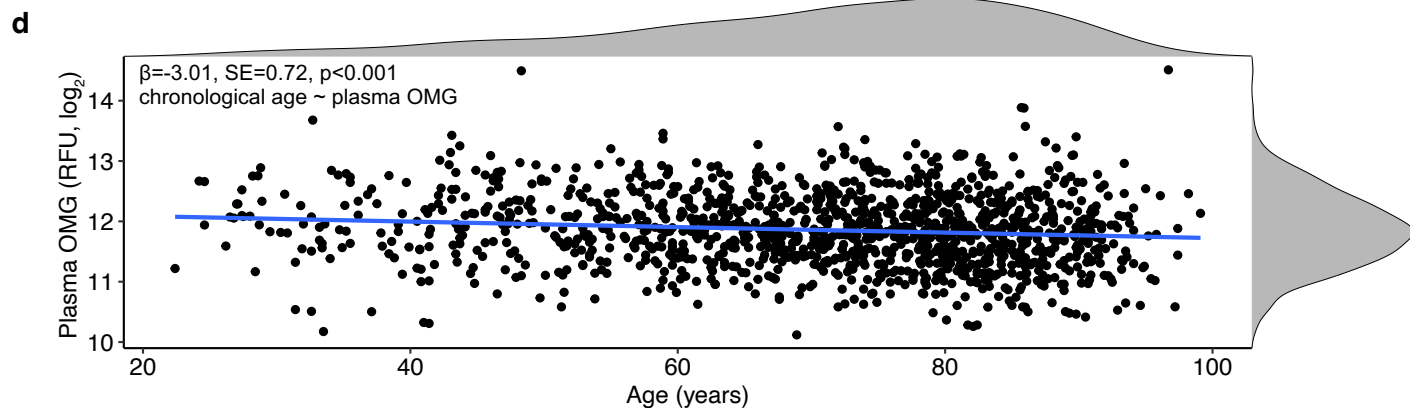

Supplement: Supplementary file 1 — Supplementary Material 1 [file 13024_2025_921_MOESM1_ESM.pdf]

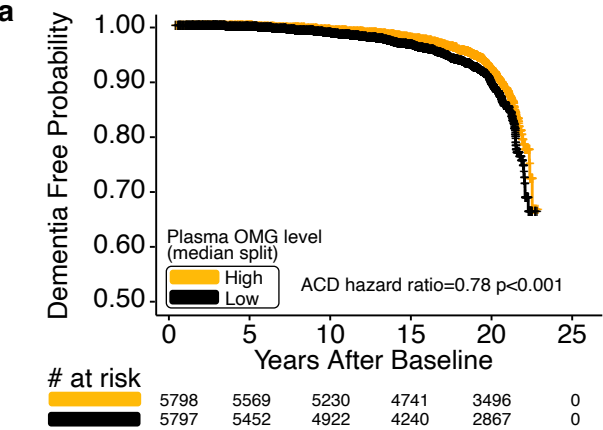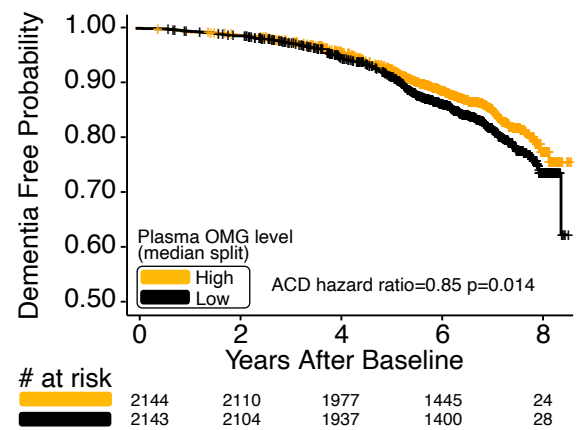

ARIC

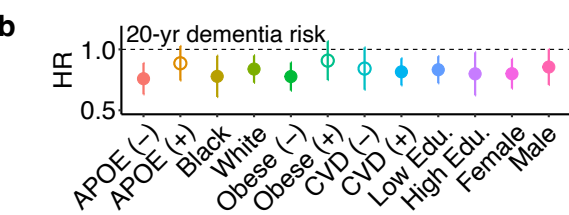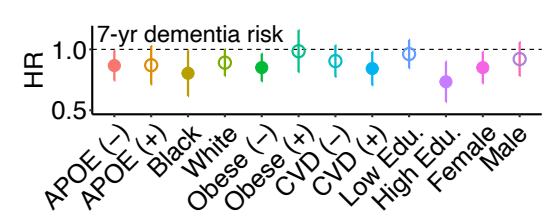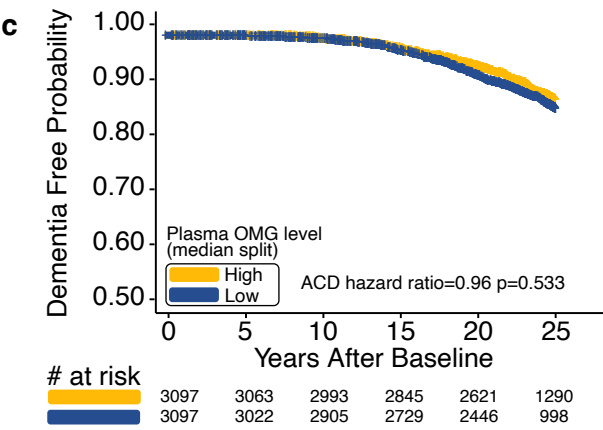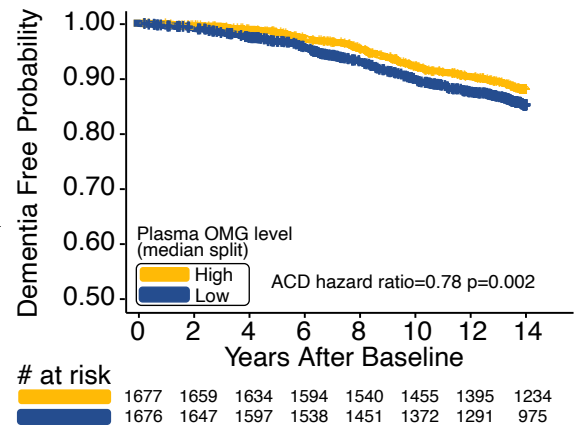

Whitehall II

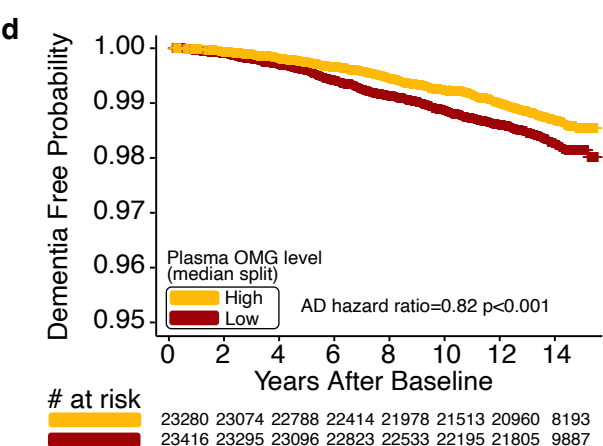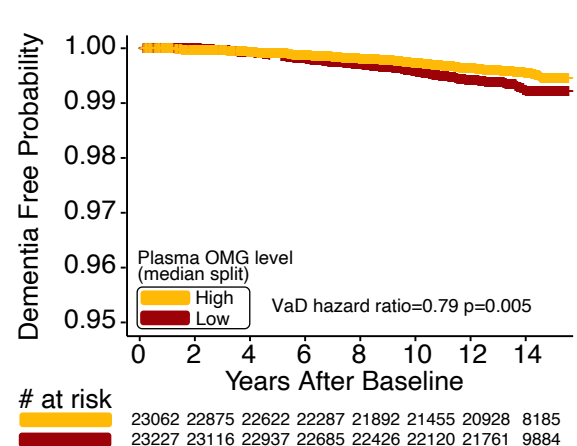

UKB

Supplement: Supplementary file 2 — Supplementary Material 2 [file 13024_2025_921_MOESM2_ESM.pdf]

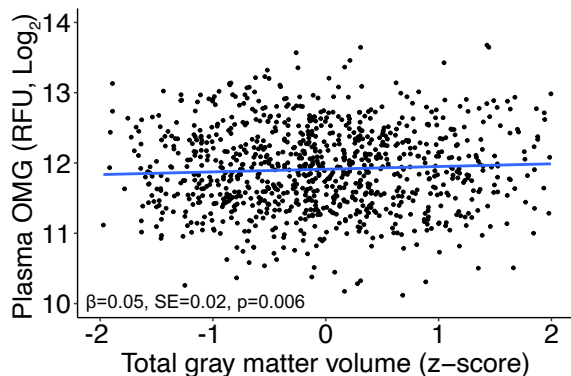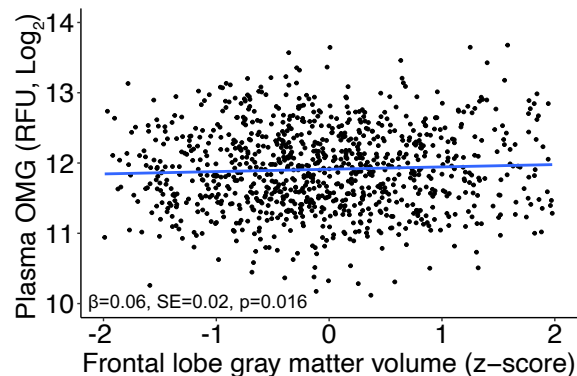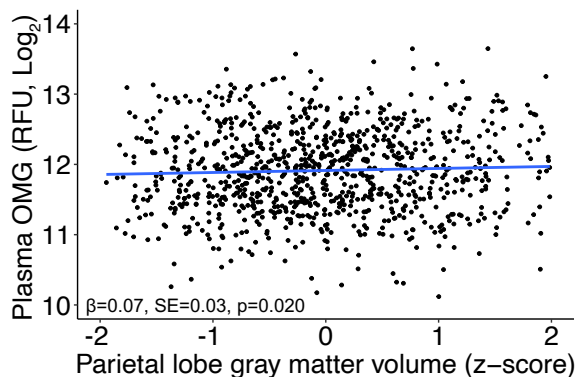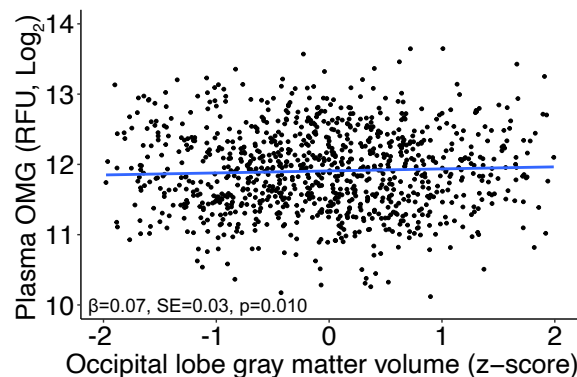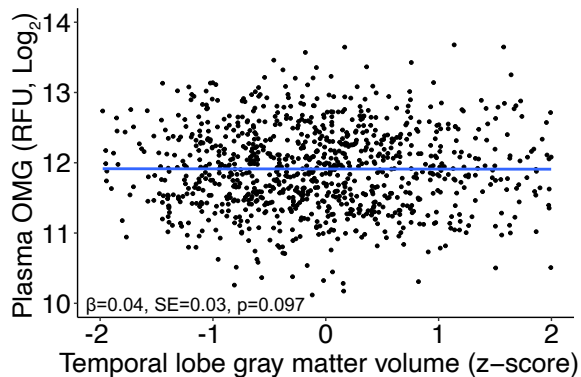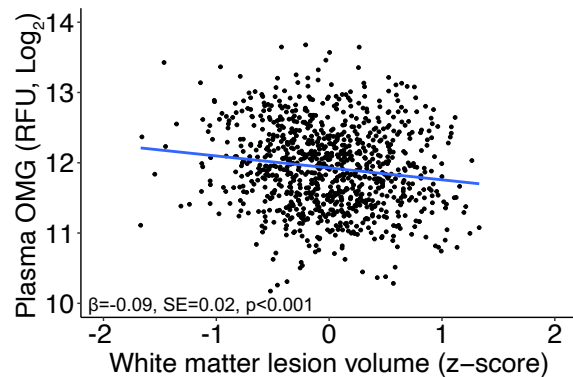

brain volume ~ plasma OMG+intracranial volume+age+sex+race+education+APOE4+comorbidity index

Supplement: Supplementary file 3 — Supplementary Material 3 [file 13024_2025_921_MOESM3_ESM.pdf]

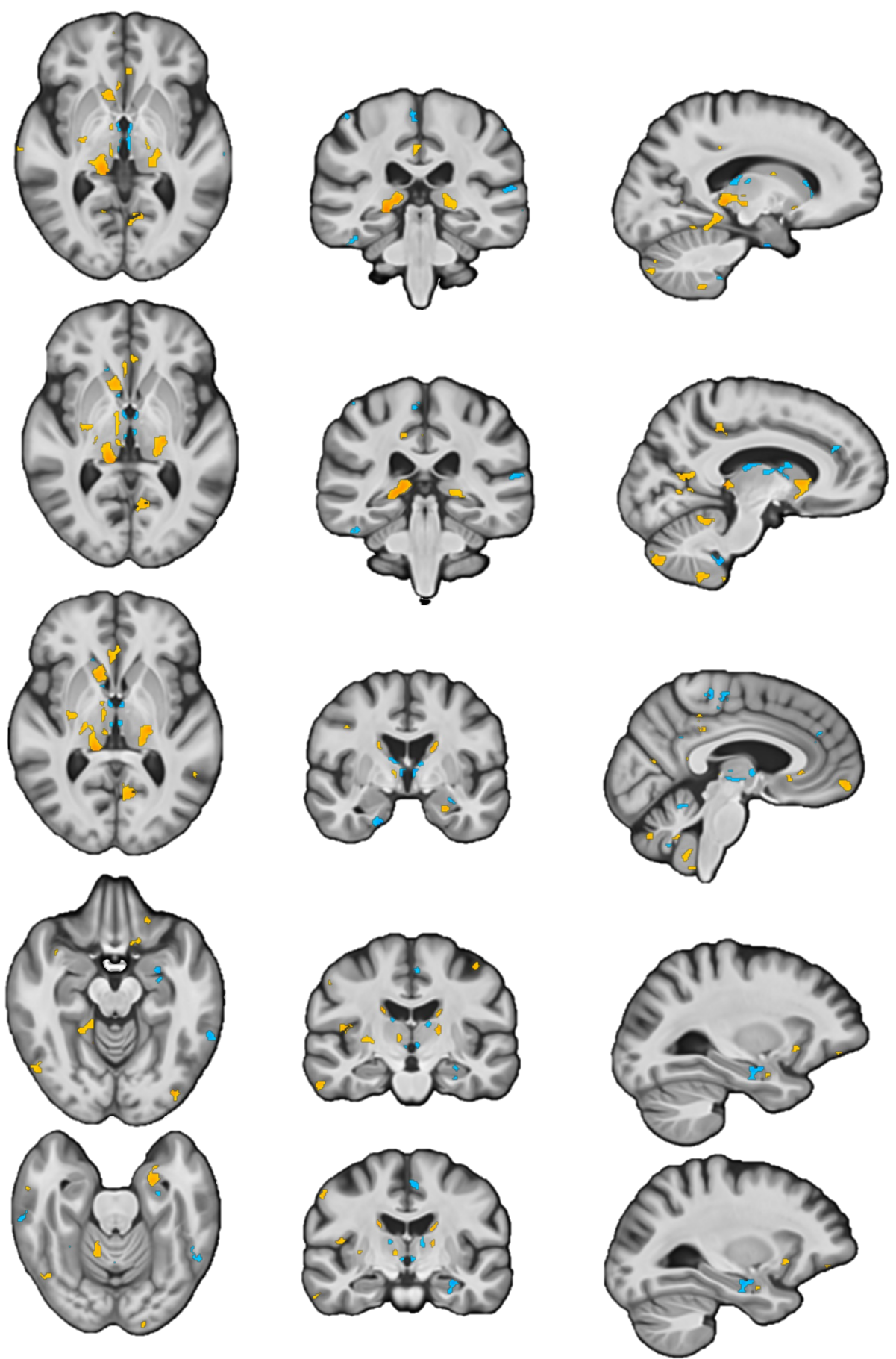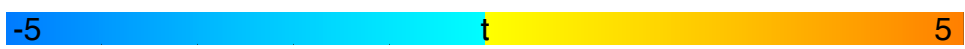

Higher brain volume →

Supplement: Supplementary file 4 — Supplementary Material 4 [file 13024_2025_921_MOESM4_ESM.pdf]

**a**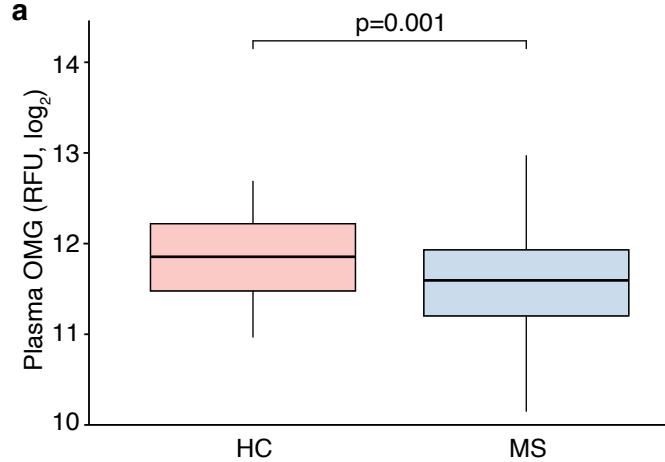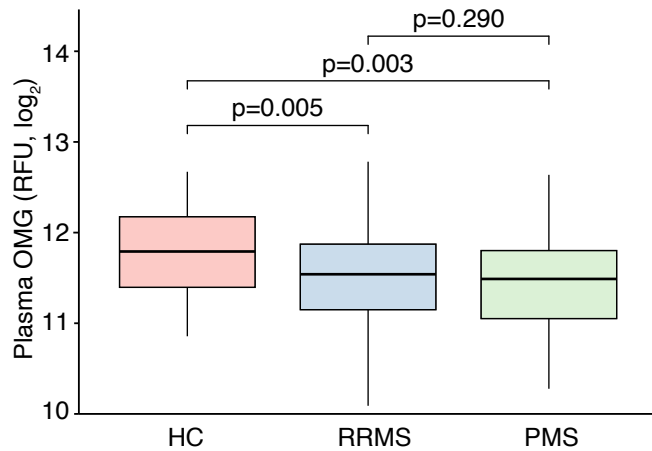**b**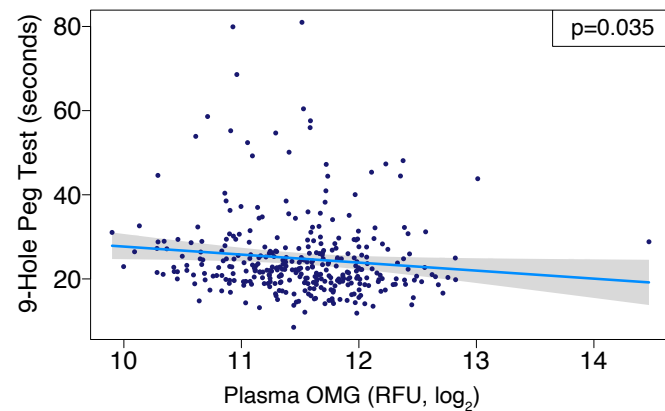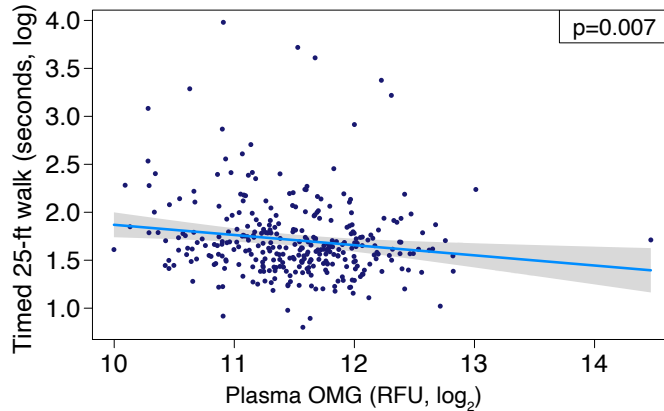

Supplement: Supplementary file 5 — Supplementary Material 5 [file 13024_2025_921_MOESM5_ESM.pdf]

**a**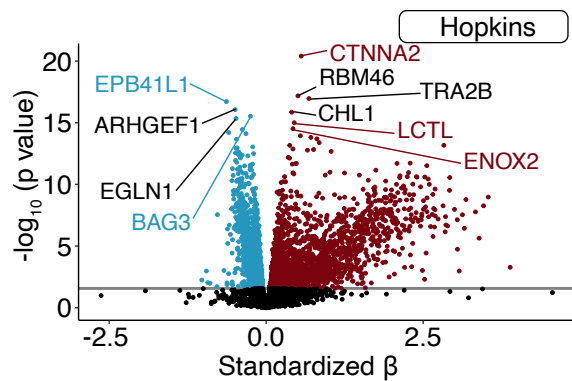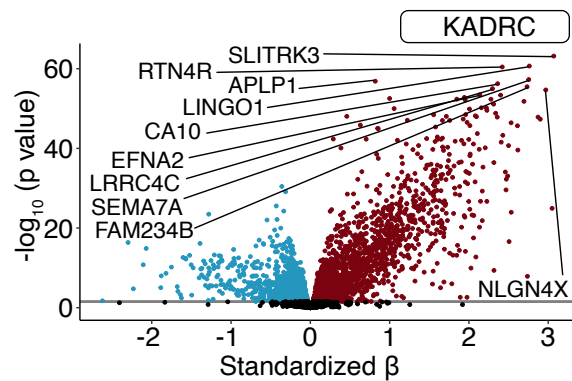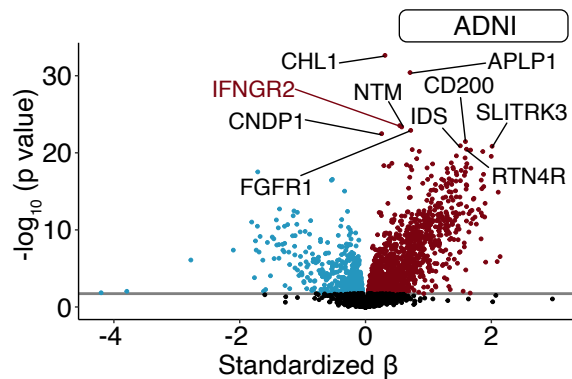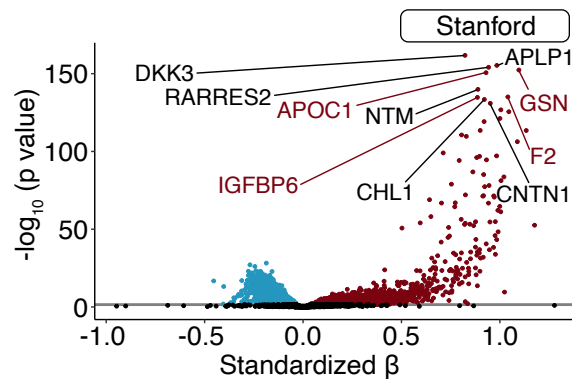**b**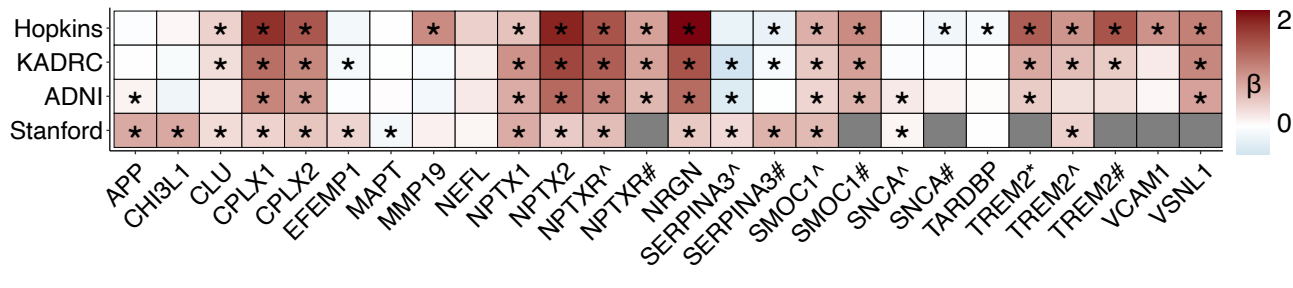

Supplement: Supplementary file 6 — Supplementary Material 6 [file 13024_2025_921_MOESM6_ESM.pdf]
