# Supplementary material for: OMG! A proteomic determinant of neurodegenerative resiliency
Source: Mol Neurodegener. 2026 Jan 5;21:9. doi: 10.1186/s13024-025-00921-1 (PMC12870269; doi:10.1186/s13024-025-00921-1)
Supplement: Supplementary file 7 — Supplementary Material 7 [file 13024_2025_921_MOESM7_ESM.pdf]

a

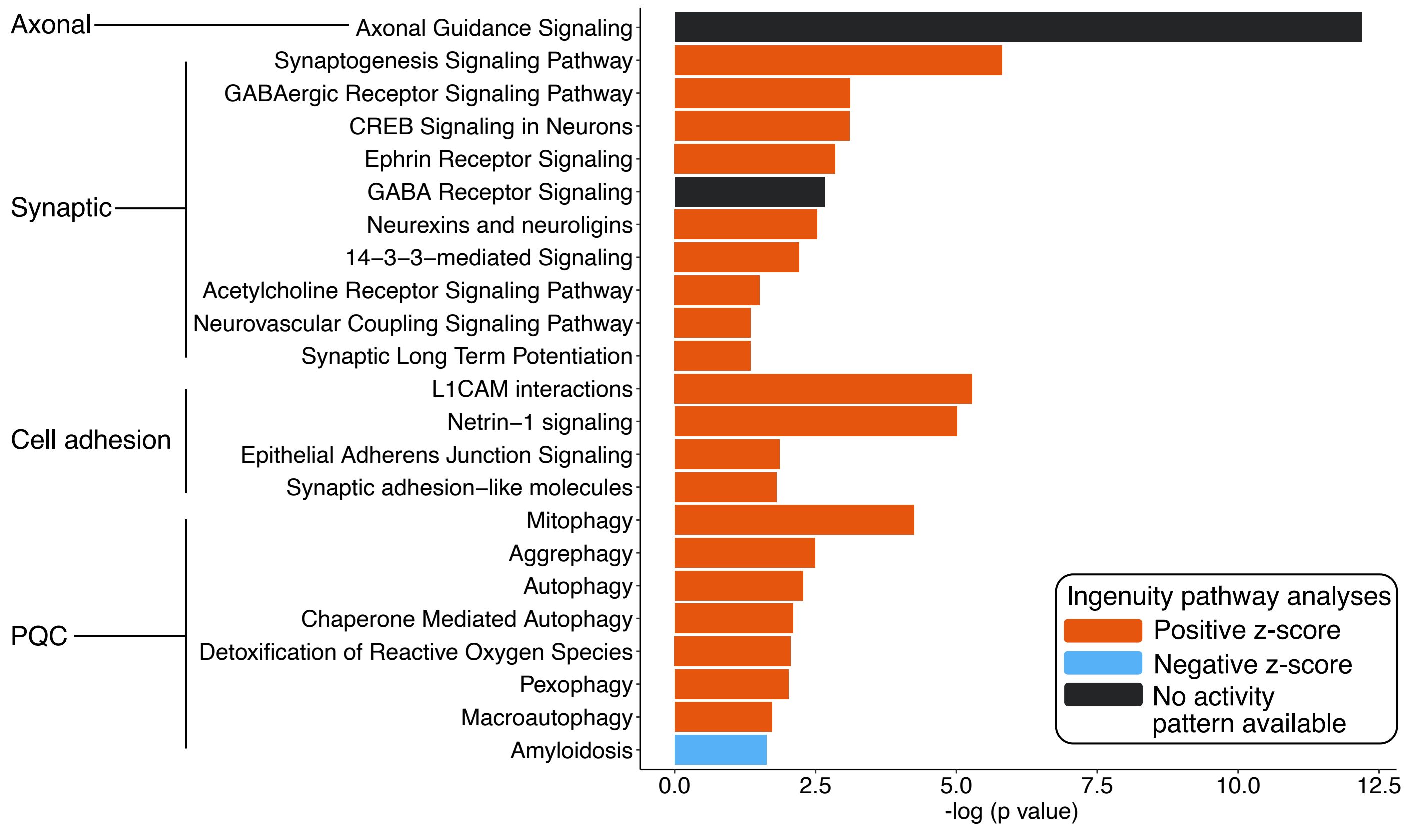

b

Database	GO Biological processes	GO Cellular component	GO Molecular function	KEGG
Top upregulated pathways	Axon guidance	Neuron projection	Transmembrane receptor protein tyrosine kinase activity	Axon guidance
	Axonogenesis	Axon	Cell adhesion mediator activity	Cell adhesion molecules
Top downregulated pathways	Positive regulation of protein phosphorylation	Focal adhesion	Zinc ion binding	Hepatitis B
	Regulation of apoptotic process	Cell-substrate junction	Receptor ligand activity	Yersinia infection
