# Supplementary material for: OMG! A proteomic determinant of neurodegenerative resiliency
Source: Mol Neurodegener. 2026 Jan 5;21:9. doi: 10.1186/s13024-025-00921-1 (PMC12870269; doi:10.1186/s13024-025-00921-1)
Supplement: Supplementary file 8 — Supplementary Material 8 [file 13024_2025_921_MOESM8_ESM.pdf]

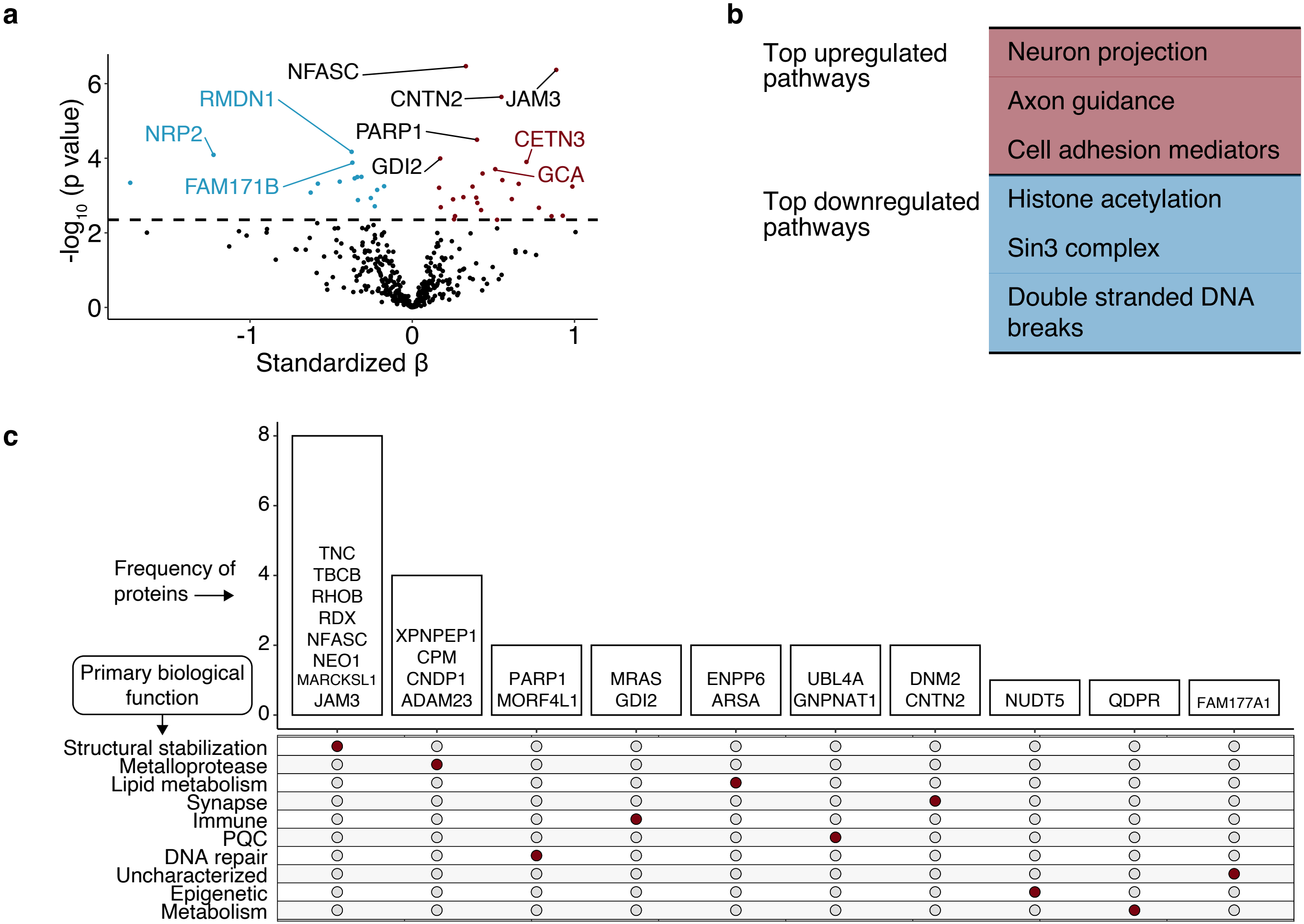
a
-log10 (p value)
Standardized β
0
2
4
6
-1
0
1
NFASC
CNTN2
JAM3
RMDN1
NRP2
PARP1
CETN3
GDI2
GCA
FAM171B
b
Top upregulated pathways
Neuron projection
Axon guidance
Cell adhesion mediators
Top downregulated pathways
Histone acetylation
Sin3 complex
Double stranded DNA breaks
c
Frequency of proteins
8
6
4
2
0
TNC
TBCB
RHOB
RDX
NFASC
NEO1
MARCKSL1
JAM3
XPNPEP1
CPM
CNDP1
ADAM23
PARP1
MORF4L1
MRAS
GDI2
ENPP6
ARSA
UBL4A
GNPNAT1
DNM2
CNTN2
NUDT5
QDPR
FAM177A1
Primary biological function
Structural stabilization
Metalloprotease
Lipid metabolism
Synapse
Immune
PQC
DNA repair
Uncharacterized
Epigenetic
Metabolism
