# Supplementary material for: OMG! A proteomic determinant of neurodegenerative resiliency
Source: Mol Neurodegener. 2026 Jan 5;21:9. doi: 10.1186/s13024-025-00921-1 (PMC12870269; doi:10.1186/s13024-025-00921-1)
Supplement: Supplementary file 9 — Supplementary Material 9 [file 13024_2025_921_MOESM9_ESM.pdf]

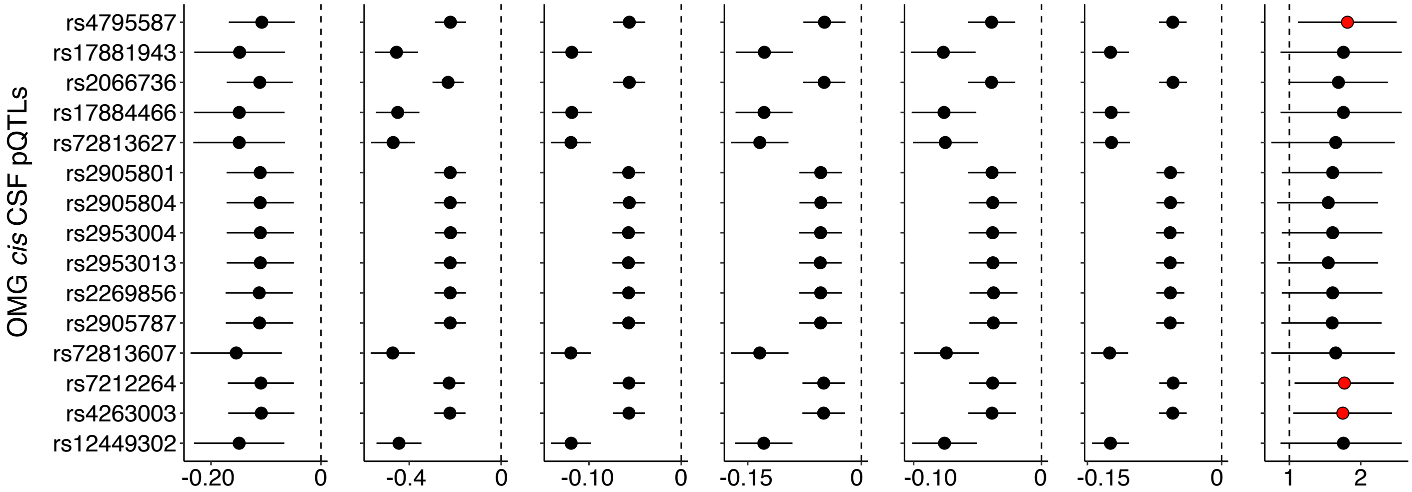


| | | | | | | | |
|---|---|---|---|---|---|---|---|
| **Statistic** | Beta | Beta | Beta | Beta | Beta | Beta | Odds ratio |
| **Outcome measure** | $\log_{10}$ OMG | $\log_{2}$ OMG | Rank-inv. OMG | Rank-inv. OMG | $\log_{2}$ OMG | $\log_{2}$ OMG | Prevalent dementia |
| **Platform** | SomaScan | Mass spec. | SomaScan | SomaScan | SomaScan | Olink | NA |
| **Specimen** | CSF | DLPFC | Plasma | Plasma | Plasma | Plasma | NA |
| **Cohort** | Wash U | ROSMAP/Banner | deCODE | Fenland | ARIC | UKB | BLSA |
