# Supplementary material for: OMG! A proteomic determinant of neurodegenerative resiliency
Source: Mol Neurodegener. 2026 Jan 5;21:9. doi: 10.1186/s13024-025-00921-1 (PMC12870269; doi:10.1186/s13024-025-00921-1)
Supplement: Supplementary file 10 — Supplementary Material 10 [file 13024_2025_921_MOESM10_ESM.pdf]

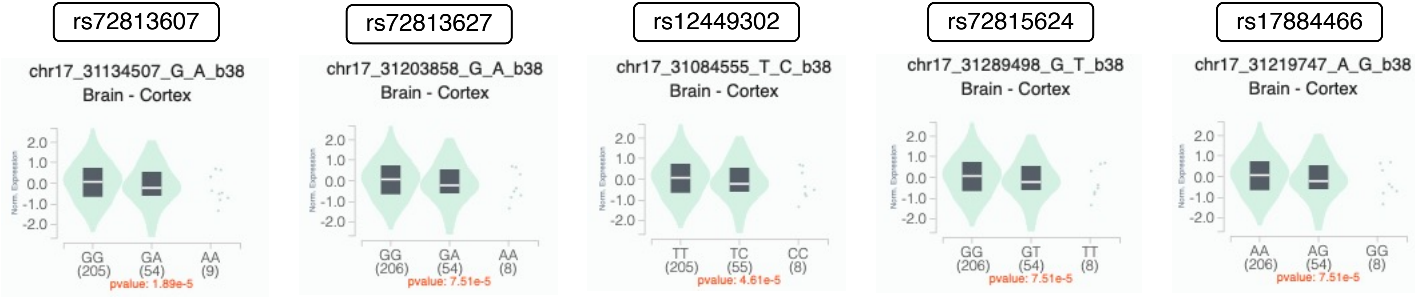
rs72813607
chr17_31134507_G_A_b38
Brain - Cortex
2.0
1.0
0.0
-1.0
-2.0
GG (205)
GA (54)
AA (9)
pvalue: 1.89e-5
rs72813627
chr17_31203858_G_A_b38
Brain - Cortex
GG (206)
GA (54)
AA (8)
pvalue: 7.51e-5
rs12449302
chr17_31084555_T_C_b38
Brain - Cortex
TT (205)
TC (55)
CC (8)
pvalue: 4.61e-5
rs72815624
chr17_31289498_G_T_b38
Brain - Cortex
GG (206)
GT (54)
TT (8)
pvalue: 7.51e-5
rs17884466
chr17_31219747_A_G_b38
Brain - Cortex
AA (206)
AG (54)
GG (8)
pvalue: 7.51e-5
