# Supplementary material for: OMG! A proteomic determinant of neurodegenerative resiliency
Source: Mol Neurodegener. 2026 Jan 5;21:9. doi: 10.1186/s13024-025-00921-1 (PMC12870269; doi:10.1186/s13024-025-00921-1)
Supplement: Supplementary file 11 — Supplementary Material 11 [file 13024_2025_921_MOESM11_ESM.docx]

**Supplementary methods**

**The Baltimore Longitudinal Study of Aging (BLSA)**

The BLSA is an ongoing study designed to assess physical and cognitive aging in a cohort of community-dwelling volunteers who primarily reside in the metropolitan area of Baltimore, MD^1^. Blood samples used for proteomic analyses were collected in 2008-2010 at the time of the first 3T MRI scan; for participants who underwent Aβ PET, proteomic analyses were conducted on samples collected at the time of their initial PET scan. Samples were collected using standard protocols and frozen at -80°C until analysis. Participants were excluded for missing covariate data. Participants with evidence of cognitive impairment concurrent with blood draws were also excluded, except for analyses that used autopsy data (due to limited sample size; n=40) or analyses that specifically examined cognitive status as an outcome.

**Plasma proteomics**

Proteins were measured using the SomaScan v4.1 platform (7,288 SOMAmer reagents), as described previously^2^. Samples from participants that did not pass SomaScan quality control (QC) criteria were excluded (n=7). Using 102 blind duplicates, proteins with intra-assay coefficient of variation (CV) >50% were excluded (n=20); OMG’s intra-assay CV was 3.5%. Values were log_2_ transformed and those beyond 5 SDs were winsorized.

**Plasma biomarkers**

Aβ_40_, Aβ_42_, GFAP, NfL and pTau-181 concentrations in plasma were measured using the Single Molecule Array (Simoa) Neurology 4-Plex E (N4PE) and pTau-181 (V2) assays. Samples were run in duplicate, and values averaged. CVs were 1.5, 1.0, 4.4, 4.9, and 4.8% for Aβ_40_, Aβ_42_, pTau-181, GFAP, and NfL, respectively. Aβ_42/40_ ratio was used in analyses and values for GFAP, NfL and pTau-181 were log_2_ transformed. Values were standardized and those beyond 5 SDs were winsorized.

**Amyloid PET**

Dynamic ^11^C-Pittsburgh compound-B (PiB) PET scans (70 min.) were collected on a GE Advance or Siemens High Resolution Research Tomograph scanner after an i.v. bolus injection of approximately 555 MBq of the radiotracer. Distribution volume ratio (DVR) images were computed using cerebellar gray matter as the reference region. Mean cortical Aβ reflected the average DVR values across the cingulate, frontal, parietal (including precuneus), lateral temporal, and lateral occipital regions, excluding the pre- and post-central gyri. Mean cortical DVR values were harmonized between the two scanners by leveraging longitudinal data available on both scanners for 79 participants. Aβ PET status (+/-) was defined based on a Gaussian mixture model threshold of 1.064 mean cortical DVR^3^.

**Brain proteomics**

Protein levels in brain tissue were measured using liquid chromatography-mass spectrometry with samples from the middle frontal gyrus, as described previosuly^4,5^. In brief, label free quantification was performed on detergent insoluble fractions of homogenates prepared from frozen post-mortem tissue. Peptides were eluted, ionized, and then detected on a hybrid LTQ XL Orbitrap mass spectrometer. Spectra were matched to a complete semi-tryptic human proteome database using the SageN Sorcerer SEQUEST 3.5 algorithm and protein quantification was performed based on extracted ion current measurements. Aβ plaque and tau neurofibrillary tangle distributions were defined using the Consortium to Establish a Registry for Alzheimer’s Disease and Braak staging criteria, respectively.

**Dementia adjudication**

Cognitive status was adjudicated by a panel of neurologists and neuropsychologists with expertise in dementia, as detailed previously^6^. In brief, participant serial clinical and neuropsychological data were reviewed at each consensus case conference if the participant had > 3 errors on the Blessed Information-Memory-Concentration test, or > 0.5 total combined score on the Clinical Dementia Rating Scale. Mild cognitive impairment (MCI) was based on Petersen’s criteria. Dementia was based on the criteria outlined by the Diagnostic and Statistical Manual of Mental Disorders, Third Edition-Revised.

**Cognitive domains**

Cognitive performance was assessed across six domains, as described previsouly^7^. Visuospatial ability was measured using a modified version of the Educational Testing Service Card Rotations Test and two Clock Drawing Tests (CDTs), where participants were asked to draw the hands and face of clocks indicating 3:25 and 11:10. Verbal memory was measured using immediate (sum of 5 learning trials) and long-delay free recall from the California Verbal Learning Test. Verbal fluency was measured using Verbal Fluency-Letters (F, A, S) and Verbal Fluency-Categories (fruits, animals, vegetables). Executive functioning was measured using Trail Making Test Part B and the Digit Span Backward subset of the Wechsler Adult Intelligence Scale-Revised. Attention was measured using Trail Making Test Part A and the Digit Span Forward subset of the Wechsler Adult Intelligence Scale-Revised. Scores of Trail Making Test Part A and Part B were first natural log transformed, z-scored and then signs inverted, so that higher scores reflect higher performance (i.e., consistent with the direction of performance of other cognitive tasks). Processing speed was measured using the Digit Symbol Substitution Task. Composite scores of visuospatial ability, verbal memory and fluency, executive functioning, and attention were calculated from standardized and averaged individual task components.

**3T MRI**

T_1_-weighted magnetization-prepared rapid gradient echo (MPRAGE) scans were acquired on a 3T Philips Achieva (repetition time [TR] = 6.8 ms, echo time [TE] = 3.2 ms, ﬂip angle = 8°, image matrix = 256 × 256, 170 slices, pixel size = 1 × 1 mm, slice thickness = 1.2 mm, sagittal acquisition). We applied a validated, Multi-atlas Region Segmentation Utilizing Ensembles (MUSE) anatomic labeling method specifically designed to achieve a consistent parcellation of brain anatomy in longitudinal MRI studies using T_1_-weighted sequences^8^. Voxel-wise maps for different brain tissue types were also calculated using the validated RAVENS methodology; a t-value threshold of 2.0 and cluster extent of 100 voxels was used to define significant clusters^9^. Regional analyses examined volumes of total brain, gray matter, white matter, and lobar regions (Frontal, Parietal, Occipital, Temporal). Diffusion tensor imaging (DTI) acquisition and image processing followed standard procedures for tensor fitting and quality assessment, as described previously in detail^10^. Each DTI session acquired two images that were concatenated and analyzed together to estimate tensors. White matter tracts were labeled using the Eve White Matter atlas to obtain average fractional anisotropy (FA) and mean diffusivity (MD) indices per tract. Using volumes of MUSE-segmented brain regions as input features, we leveraged a semi-supervised representation learning via generative adversarial networks approach (Surreal-GAN) to calculate R indices (R1, R2, R3, R4, R5). By distinguishing heterogenous brain volume differences between younger (<50 years old) and older (>50 years old) adults, this approach generates multiple, continuous, low-dimensional scores that reflect the co-expression level of respective brain atrophy dimensions, and accounts for simultaneous spatial and temporal disease heterogeneity within the same individual. These scores have been pretrained and validated in a diverse cohort across 11 studies (>49,000 participants), where they predicted age-related clinical traits and disease diagnoses^11^. Along with the T_1_-weighted images, T_2_-weighted fluid attenuated inversion recovery (FLAIR; TR = 11 s, TE = 68 ms, inversion time = 2800 ms, image matrix = 240 × 240 × 150, voxel size = 0.83 mm×0.83 mm×3 mm) scans were used to segment white matter hyperintensities with a convolutional deep neural network (DeepMRSeg)^12^. This pretrained deep learning segmentation method utilizes a modified UNet architecture that takes advantage of multiple convolution filter sizes to achieve multi-scale feature extraction adaptive to the desired segmentation task, and has been validated on a wide range of extraction tasks, including white matter lesion, deep brain structure, and hippocampal formation data. White matter hyperintensity volumes were log transformed.

**Genetics**

Genome-wide genotyping in the was performed using the Illumina 550K or NeuroChip platforms using standard QC procedures described previously^13^. Variants were excluded for poor call rate (missing>1%), violations of Hardy-Weinberg Equilibrium (p<1x10^-6^) and limited minor allele frequency (<1%). Samples were excluded for poor genotyping efficiency (missing>2%), sex inconsistencies (i.e., discordance between chromosomal and self-reported sex), or cryptic relatedness (pihat>0.25). Imputation was performed for Illumina 550K or NeuroChip datasets separately with the Michigan Imputation Server MaCH (https://imputationserver.sph.umich.edu/) using the HRC r1.1.2016 reference panel. After imputation, datasets were merged, overlapping samples were removed, and SNPs with low imputation quality (*R*^2^< 0.9), minor allele frequencies <1%, Hardy-Weinberg Equilibrium p-values<1x10^-6^ , and those that did not overlap between the 2 datasets (missingness<99%) were excluded. The final genetic dataset included 5,439,477 SNPs.

**Covariates**

Age (years), sex (male/female), race (white/non-white), and education level (years) were defined based on self-report. APOE4 carrier status (0 ε4 alleles/≥1 ε4 alleles/missing) was defined via PCR with restriction isotyping using the Type IIP enzyme Hhai or a Taqman assay. All other covariates were quantified concurrently with plasma proteomic measurements. Comorbid diseases that represent potential confounders were defined using a comorbidity index calculated as the sum (score range: 0-8; converted to a percentage to account for missing data) of eight conditions: obesity, hypertension, diabetes, cancer, ischemic heart disease, chronic heart failure, chronic kidney disease and chronic obstructive pulmonary disease^14^.

**Atherosclerosis Risk in Communities (ARIC) study**

ARIC is an ongoing, community-based study that enrolled 15,792 mostly White and Black participants from four US communities (Jackson, MS; northwestern suburbs of Minneapolis, MN; Forsyth County, NC; and Washington County, MD) between 1987 and 1989^15^. Until Visit 4 (1996–1998), participants were evaluated every 3 years at in-person study visits. In-person Visit 5 was conducted in 2011–2013, Visit 6 in 2016–2017, and Visit 7 in 2018-2019. Data collection for subsequent ARIC study visits is ongoing. Blood samples for proteomic analyses were collected at Visit 2 and Visit 5 using standard protocols and frozen at -80°C until analysis. Participants were excluded for missing covariate data, if they were non-White or non-Black participants, Black participants from Minneapolis and Washington Counties (due to low sample sizes), or had a dementia diagnosis on or before the date of blood sample collection (Visit 2 for mid-life analyses, Visit 5 for late-life analyses).

**ARIC plasma proteomics**

Proteins were measured using the SomaScan v4.0 platform (4,971 SOMAmer reagents), as previously described^16^. Using 187 blind duplicates, proteins with intra-assay CV >50% were excluded (n=94); OMG’s CV was 4.0% at Visit 2 and 6.6% at Visit 5. Samples from participants that did not pass QC criteria were excluded (n=15). Values were log_2_ transformed and those beyond 5SDs were winsorized.

**ARIC plasma biomarkers**

Aβ_40_, Aβ_42_, GFAP, NfL and pTau-181 concentrations were measured using the Single Molecule Array (Simoa) Neurology 4-Plex E (N4PE) and pTau-181 (V2) assays on the Simoa HD-X instrument (Quanterix)^17^. Using 90 blind duplicates, CVs were 7.3, 8.5, 2.3, 3.7 and 5.6% for Aβ_40_, Aβ_42_, GFAP, NfL and pTau-181, respectively. Values for GFAP, NfL and pTau-181 were log_2_ transformed to correct for skewness, and Aβ_42/40_ ratio were used in analyses.

**ARIC amyloid PET**

^18^F-florbetapir standard uptake value ratios (SUVR; 20 min.) were collected at ARIC Visit 5 on Siemens scanners (various models across ARIC sites) 50-70 min. after an i.v. bolus injection of the radiotracer, as described previously^18^. SUVRs were calculated using cerebellar gray matter region as a reference. Mean cortical Aβ reflected average SUVR values across the orbitofrontal, prefrontal, and superior frontal cortices, lateral temporal, parietal, and occipital lobes, precuneus, and anterior and posterior cingulate regions of interest. Aβ status (+/-) was defined based on a median of 1.20 mean cortical SUVR.

**ARIC dementia adjudication**

Mid-life dementia risk was assessed between Visits 2-5, and late-life dementia risk was assessed between Visits 5-7^19^. Dementia surveillance between enrollment and Visit 5 have been detailed previously^20,21^. At Visits 2 and 4, participants were administered three neurocognitive tests (delayed word recall, digit symbol substitution, and word fluency test). For a subset of participants suspected of having dementia, a modified version of the Clinical Dementia Rating (CDR) scale and the Functional Activities Questionnaire (FAQ) were administered to informants. For participants who attended Visit 5, these measures were used to estimate the date of dementia onset. For participants who did not attend Visit 5 (due to death or non-attendance), the Telephone Interview for Cognitive Status-Modified (TICSm), CDR, FAQ, hospital discharge codes, and death certificate codes were used to define dementia diagnosis and date of dementia onset. At Visits 5, 6, and 7, participants underwent comprehensive cognitive and functional in-person assessments to quantify memory, language, processing speed, and executive function, as described previously^21^. Between these visits, a semi-annual phone surveillance approach was implemented, where participants were annually administered the Six Item Screener (SIS), a brief cognitive assessment. If the participant received a low SIS score (or was unable to participate in the screening via phone), the Ascertain Dementia 8 (AD8) was administered to the participant’s informant. For participants who attended Visits 6 and 7, these measures were used to estimate the date of dementia onset. For participants who did not attend Visits 6 and 7 (due to death or nonattendance), these measures along with hospital discharge codes and death certificate codes were used to define dementia diagnoses and date of dementia onset. An algorithmic dementia diagnosis was initially defined when the following criteria were met: a score >5 on the FAQ or a CDR sum of boxes score >3; two or more cognitive domain scores >1.5SD below the normative mean; and previous evidence of decline on the cognitive battery of >0.055CD per year, which approximates the rate of cognitive decline in cognitively healthy older adults. All dementia diagnoses identified using the algorithm were confirmed by an expert committee of physicians and neuropsychologists based on the criteria outlined by the NIA-Alzheimer’s Association (NIA-AA), and the Diagnostic and Statistical Manual of Mental Disorders, Fifth Edition.

**ARIC 3T MRI**

T1-weighted MPRAGE scans were acquired at ARIC Visit 5 on Siemens scanners (Verio [MD study center], Skyra [NC study center], Trio [MN study center], and Skyra [MS study center]). FreeSurfer was used for anatomic labeling^22^. Regional analyses examined volumes of total brain, hippocampal, and lobar regions (Frontal, Parietal, Occipital, Temporal). We also examined a temporal-parietal lobe composite ROI comprised of regions susceptible to but not indicative of AD (i.e., combined volumes of the hippocampus, precuneus, parahippocampal gyrus, entorhinal cortex, and inferior parietal lobes), a composite ROI of deep gray subcortical structures (i.e., combined volumes of the thalamus, caudate, putamen, and globus pallidum), and cortical thickness^23^. DTI acquisition and image processing followed standard procedures for tensor fitting and quality assessment, as described previously in detail^24^. Segmentation used an in-house atlas based on the STAND400 template. FA and MD were calculated from a set of commissural (genu, body, and splenium of the corpus callosum), association (superior fronto-occipital fasciculus), and projection (posterior limb of the internal capsule) tracts. Cerebral microhemorrhages and lacunar infarcts were identified, counted, and measured by a trained imaging technician and confirmed by radiologists, as previously described. T2-weighted FLAIR scans were used to segment white matter hyperintensities with computer-aided segmentation program (FLAIR-histoseg)^25^; white matter hyperintensity volumes were log transformed.

**ARIC covariates**

Participant education (less than high school/high school; general education diploma or vocational school/at least some college), race (Black/White), and sex (male/female) were reported at enrollment. Because race and study center are highly confounded, a race-study center variable was used (White-Washington County/White-Forsyth County/Black-Forsyth County/White-Minneapolis/Black-Jackson). APOE4 carrier status (0 ε4 alleles/≥1 ε4 alleles/missing) was defined via PCR with a Taqman assay. All other covariates were quantified concurrently with plasma proteomic measurements.

**Emory Goizueta AD Research Center (Emory-ADRC)**

We leveraged publicly available data from 18 control and 18 AD participants in the Emory-ADRC^26^. The Emory-ADRC is a multidisciplinary center research, diagnosis, and treatment center for AD and related disorders; it operates from the Emory University School of Medicine (with additional support from the Emory Healthy Brain Study and Emory Cognitive Neurology Clinic) in the metropolitan area of Atlanta, GA. Blood samples were collected concurrently with outcome measurements (e.g., cognitive assessment) using standard protocols and frozen at -80°C until analysis. Blood and CSF samples were collected at or near the same time point for each participant. Participants were excluded for missing covariate data.

**Emory-ADRC plasma proteomics**

Proteins were measured using the SomaScan v4.1 platform (7,288 SOMAmer reagents) and Olink Explore platform (1,196 targets), as previously described^26^. All samples passed SomaScan and Olink QC criteria. Proteins with >75% missingness were excluded (n=4 SomaScan; n=56 Olink). Samples with measurements below assay-specific limits of detection (calculated as median log_2_ buffer signal plus 3SDs) were considered missing; a single Olink sample measurement of OMG from one participant met this criteria. SomaScan values were log_2_ transformed and those beyond 5 SDs were winsorized. Olink values were retained as log_2_ normalized protein expression.

**Emory-ADRC CSF proteomics**

Proteins were measured using the SomaScan v4.1 platform (7,288 SOMAmer reagents), as described above. All samples passed SomaScan QC criteria. Proteins with >75% missingness were excluded (n=3,694). Protein measurements below assay-specific limits of detection (calculated as median log_2_ buffer signal plus 3SDs) were considered missing. SomaScan values were log_2_ transformed and those beyond 5 SDs were winsorized.

**Emory-ADRC CSF biomarkers**

Aβ_42_, total tau, and pTau-181 were measured with INNO-BIA AlzBio3 Luminex assay, as described previously^27^.

**Emory-ADRC AD diagnosis**

Diagnoses were adjudicated based on criteria outlined by the NIA-AA.

**Emory-ADRC covariates**

APOE4 carrier status (0 ε4 alleles/≥1 ε4 alleles) was defined via PCR with an Affymetrix or a Taqman assay. All other covariates were quantified concurrently with biofluid proteomic measurements.

**Stanford cohort**

The Stanford cohort consisted of participants enrolled in four studies, the Iqbal Farrukh and Asad Jamal Stanford AD Research Center (SADRC), the Stanford Aging and Memory Study (SAMS), the Stanford Biomarkers in PD Study (BPD), and the Stanford Center for Memory Disorders Cohort Study (SCMD). Participants were primarily recruited from the metropolitan area of San Francisco, CA. The SADRC is a longitudinal observational study of clinical dementia subjects and cognitively normal (age-sex-matched) subjects. SAMS is an ongoing longitudinal study of functional and structural imaging, biofluid, genetic, and cognitive features in healthy aging and preclinical AD. The BPD was a Michael J. Fox Foundation for Parkinson’s Research funded longitudinal study of biological markers associated with cognitive decline. The SCMD was an NIA-funded cross-sectional study of people across the cognitive continuum. Biofluid samples were collected concurrently with outcome measurements (e.g., cognitive assessment) using standard protocols and frozen at -80°C until analysis. Participants were excluded for missing covariate data.

**Stanford plasma proteomics**

Proteins were measured using the SomaScan v4.1 platform (7,288 SOMAmer reagents), as described previously^28^. Samples from participants that did not pass SomaScan QC criteria were excluded. Proteins with high CVs and low assay-version stability were excluded (n=201). OMG’s estimated CV was 4.0%. Values were log_2_ transformed.

**Stanford CSF proteomics**

Proteins were measured using the SomaScan v4.0 platform (4,971 SOMAmer reagents), as described previously^29^. Samples from participants that did not pass SomaScan QC criteria were excluded. Values were log_2_ transformed.

**Stanford dementia adjudication**

Cognitive status was adjudicated in a clinical consensus conference that included expert neurologists and neuropsychologists. Standardized assessments, including the CDR, were used to determine cognitive capacities. Diagnoses were based on criteria outlined by the NIA-AA.

**Stanford 3T MRI**

T1-weighted magnetization-prepared rapid gradient echo (MPRAGE) scans were acquired on a GE Discovery scanner (MR750; TR = 7.26 ms, FoV = 230 mm × 230 mm, voxel size = 0.9 × 0.9 × 0.9 mm, slices = 186). FreeSurfer was used for anatomic labeling. Regional analyses examined total gray matter, subcortical gray matter, cerebral white matter, cortical, and hippocampal volumes.

**Stanford covariates**

APOE4 carrier status (0 ε4 alleles/≥1 ε4 alleles) was determined using allelic combinations of single nucleotide variants rs7412 and rs429358. All other covariates were quantified concurrently with biofluid proteomic measurements.

**Religious Orders Study/Rush Memory and Aging Project (ROSMAP)**

ROSMAP consists of two continuously enrolling community based cohort studies, ROS across the USA and MAP across northeastern Illinois^30^. Participants enrolled without known dementia and agreed to annual detailed clinical evaluations. Study visits employed complete clinical and functional evaluations, including medical history reports, cognitive assessments, and blood draws. Blood samples were collected concurrently with outcome measurements (e.g., cognitive assessment) using standard protocols and frozen at -80°C until analysis. Participants were excluded for missing covariate data.

**ROSMAP plasma proteomics**

Proteins were measured using the SomaScan v4.1 platform (7,288 SOMAmer reagents), as described previously^29^. Samples from participants that did not pass SomaScan QC criteria were excluded (n=73). Values were log_2_ transformed.

**ROSMAP dementia adjudication**

Cognitive status was adjudicated using a multi-stage process^31^. First, participants completed a comprehensive cognitive testing battery, where education-adjusted cutoff scores were used to define impairment across five cognitive domains. To give a clinical judgement on domain-specific cognitive impairment, neuropsychologists reviewed impairment classifications, other cognitive test results, as well as information on education, occupation, sensory and motor deficits and comments from neuropsychological testing. This classification was then integrated with all other available participant information obtained from comprehensive clinical and functional evaluations, including self-reported history of cognitive decline. A neurologist, geriatrician, or geriatric nurse practitioner with expertise in dementia reviewed all participant information and provided a final diagnosis.

**ROSMAP covariates**

APOE4 carrier status (0 ε4 alleles/≥1 ε4 alleles) was determined using high-throughput sequencing of codons 112 and 158 in APOE (Agencourt Bioscience Corporation). All other covariates were quantified concurrently with plasma proteomic measurements.

**Knight AD Research Center (Knight-ADRC)**

The Charles and Joanne Knight AD Research Center (ADRC) is an NIA-funded longitudinal observational study of clinical dementia subjects and age-matched controls^32^. Knight-ADRC participants were primarily recruited from the metropolitan area St. Louis, MO, and undergo longitudinal cognitive, neuropsychologic, neuroimaging, and biomarker assessments. Biofluid samples were collected concurrently with outcome measurements (e.g., cognitive assessment) using standard protocols and frozen at -80°C until analysis. Participants were excluded if they had evidence of non-AD neurodegenerative diseases or missing covariate data.

**Knight-ADRC plasma proteomics**

Proteins were measured using the SomaScan v4.1 platform (7,288 SOMAmer reagents), as described previously^33^. Proteins with >15% missingness were excluded. Protein measurements below SomaScan limit of detection (calculated as average buffer signal plus 2SDs), with a CV >15%, with a scale factor difference (calculated as the absolute value of the maximum difference between the calibration scale factor per aptamer and the median for each of the plates run) >0.5, or with a log_10_ value +/-1.5IQR were considered missing. Samples from participants with >15% missingness were excluded. Values were log_2_ transformed.

**Knight-ADRC CSF proteomics**

Proteins were measured using the SomaScan v4.1 platform (7,288 SOMAmer reagents), as described above. Proteins and samples with >15% missingness were excluded. Values were log_2_ transformed.

**Knight-ADRC dementia adjudication**

Cognitive status was adjudicated by a neurologist with expertise in dementia. Standard assessments, including the CDR, were used to determine cognitive capacities. Diagnoses were based on criteria outlined by the National Institute of Neurological and Communicative Disorders and Stroke-Alzheimer's Disease and Related Disorders Association (NINDS-ADRDA).

**Knight-ADRC covariates**

APOE4 carrier status (0 ε4 alleles/≥1 ε4 alleles/missing) was defined via PCR with a Taqman assay. All other covariates were quantified concurrently with biofluid proteomic measurements.

**AD Neuroimaging Initiative (ADNI)**

ADNI is a public-private consortium of universities and medical centers across the United States and Canada. This longitudinal, multi-center study uses MRI, PET, and other biomarker data to develop uniform data acquisition standards, a publicly available data repository, and biomarker methods for early AD detection. CSF samples were collected concurrently with outcome measurements (e.g., cognitive assessment) using standard protocols and frozen at -80°C until analysis. Participants were excluded if they had evidence of non-AD neurodegenerative diseases or missing covariate data.

**ADNI CSF proteomics**

Proteins were measured using the SomaScan v4.1 platform (7,288 SOMAmer reagents), as described previously^33^. Proteins with >15% missingness were excluded. Protein measurements below SomaScan limit of detection (calculated as average buffer signal plus 2SDs), with a CV >15%, with a scale factor difference (calculated as the absolute value of the maximum difference between the calibration scale factor per aptamer and the median for each of the plates run) >0.5, or with a log_10_ value +/-1.5IQR were considered missing. Samples from participants with >15% missingness were excluded. Values were log_2_ transformed.

**ADNI dementia adjudication**

Cognitive status was adjudicated by physicians and specialists of the ADNI Clinical Core with expertise in dementia. **E**ducation-adjusted cutoff scores of the Mini-Mental State Examination, CDR, and Logical Memory II subscale of the Wechsler Memory Scale–Revised were used to determine cognitive capacities. Diagnoses were based on criteria outlined by the NINDS-ADRDA.

**ADNI covariates**

APOE4 carrier status (0 ε4 alleles/≥1 ε4 alleles/missing) was defined via PCR with a Taqman assay. All other covariates were quantified concurrently with CSF proteomic measurements.

**The National Institute for Longevity Sciences-Longitudinal Study of Aging (NILS-LSA)**

NILS-LSA is a population-based, prospective cohort study in Aichi Prefecture, Japan that enrolled 2,267 randomly selected men and women aged 40-79 years old between 1997 through 2000. Follow-up clinical evaluations occurred every two years, which included neuropsychological assessments. Blood samples for proteomic analyses were collected at the second study visit (Wave 2; 2000-2002) using standard protocols and frozen at -80°C until analysis. Blood samples for the current analyses were randomly selected using a case-cohort approach from the whole NILS-LSA cohort (n=340; event/dementia cases n=170, non-event/non-dementia cases n=170).

**NILS-LSA plasma proteomics**

Proteins were measured using the SomaScan v4.1 platform (7,268 SOMAmer reagents), as described previously^34^. All samples passed SomaScan assay QC criteria. Values were log_2_ transformed.

**NILS-LSA dementia diagnosis**

Dementia risk was assessed between enrollment and 2022.Dementia diagnosis was adjudicated in NILS-LSA through Long Term Care Insurance, a mandatory form of national social insurance to assist activities of daily living in the disabled elderly in Japan. The dementia criteria use a scale-based adjudication, requiring information from questionnaires (developed by the Japanese Ministry of Health, Labor, and Welfare) to assess the degree of functional disability, and official documentation based on ascertainment of an attending physician (the Doctor’s Opinion Paper). According to the Levels of Independence Degree in Daily Living for Elderly with Dementia reported by the attending physician, the applicant's independence is classified into six ranks (0, I–IV, and M). Consistent with prior studies, individuals with a dementia scale degree of ≥IIa were classified as dementia cases^35^.

**NILS-LSA**

Covariates were quantified concurrently with plasma proteomic measurements.

**NILS-LSA covariates**

APOE4 carrier status (0 ε4 alleles/≥1 ε4 alleles/missing) was defined using the Type IIP enzyme Hhai. All other covariates were quantified concurrently with plasma proteomic measurements.

**Whitehall II**

Whitehall II is an ongoing, observational cohort study that enrolled 10,308 civil servants aged 35 to 55 from London, UK between 1985 and 1988^36^. Subsequent data collections occurred every one to five years, with some waves taking multiple years to complete: 1989-1990 (Phase 2), 1991-1994 (Phase 3), 1995-1996 (Phase 4), 1997-1999 (Phase 5), 2001 (Phase 6), 2002-2004 (Phase 7), 2006 (Phase 8), 2007-2009 (Phase 9), 2011 (Phase 10), 2012-2013 (Phase 11), 2015-2016 (Phase 12), 2019-2020 (Phase 13). All data collections included a questionnaire survey, while clinical examinations were conducted during Phases 3, 5, 7, 9, 11, 12, and 13. Blood samples for proteomic analyses were collected from a random sample of participants when neuropsychological assessments were initiated at Phase 5 and again at Phase 9 using standard protocols and frozen at -80°C until analysis. Participants were excluded for missing covariate data or if they had a dementia diagnosis on or before the date of blood sample collection.

**Whitehall II plasma proteomics**

Proteins were measured using the SomaScan v4.0 platform (4,971 SOMAmer reagents) and v4.1 platforms (7,268 SOMAmer reagents), as described previously^37^. 37% and 63% of proteomic data were derived from the v4.0 and v4.1 platforms, respectably. Samples from participants that did not pass SomaScan QC criteria were excluded. Values were log_2_ transformed.

**Whitehall II cognitive decline**

Mid-life cognitive decline was assessed between Phases 5-12, and late-life cognitive decline was assessed between Phases 9-12. Cognitive performance was assessed at Phase 5, 7, 9, 11, and 12 as previously described^38^ using a battery of tasks that reflect four domains (executive functioning, memory, phonemic fluency, semantic fluency) and a global cognitive score derived from these domains. Executive function was measured with the Alice Heim 4-I test; this 10-minute assessment consists of 65 increasingly difficult verbal and mathematical reasoning items that tests a participant’s ability to identify patterns and infer principles/rules. A 20-word free recall test assessed memory; here, participants were presented a list of one or two syllable words at two-second intervals and then asked to recall in writing as many of the words as possible in any order within two minutes. Phonemic fluency was tested by asking participants to recall as many words as possible beginning with “s” over the course of one minute. Semantic fluency was tested by asking participants to recall as many animal names as possible over the course of one minute. A global composite score was calculated as the sum of standardized (converted to a z-score using the baseline mean and SD) task scores.

**Whitehall II dementia diagnosis**

Mid-life dementia risk was assessed between Phases 5-13, and late-life dementia risk was assessed between Phases 9-13. Dementia was ascertained with linked electronic health records from the National Health Services Hospital Episode Statistics database and the Mental Health Service Data Set as well as death records from the National Health Services Central Registry. Dementia was ascertained using ICD9 (290.0–290.4, 331.0–331.2, 331.82, 331.9) and ICD10 (F00, F01, F03, G30, G31) codes. The first record of dementia diagnosis was used to define date of dementia onset.

**Whitehall II covariates**

Covariates were quantified concurrently with plasma proteomic measurements.

**UK Biobank (UKB)**

UKB is a population-based biomedical database and research study containing in-depth genetic, health, and lifestyle data from over 500,000 UK participants who were enrolled between 2006-2010. Blood samples for proteomic analyses were collected at enrollment using standard protocols and frozen at -80°C until analysis. Participants were excluded for missing covariate data or if they had a dementia diagnosis on or before the date of blood sample collection.

**UKB plasma proteomics**

Proteins were measured using the Olink Explore (2,923 targets), as described previously^39^. Proteomic data pre-processing followed standard UKB QC procedures, where normalized protein expression values below the lower limits of detection were retained. Using 1,474 blind duplicates, OMG’s CV was 7.0%. Olink values were retained as log_2_ normalized protein expression.

**UKB 3T MRI**

T1-weighted MPRAGE scans were acquired on a 3-T Siemens Skyra as previously described^40^, and machine learning-derived neuroimaging measures of age- and AD-related brain atrophy were generated using the same methodology as described in the BLSA.

**UKB dementia diagnoses**

Etiology-specific dementia risk was assessed between enrollment and 2022. Dementia was ascertained with linked hospital discharge records, primary care records, and death certificates using diagnostic codes for all-cause dementia, Alzheimer’s disease, Vascular dementia, Frontotemporal dementia, and Parkinson’s disease dementia; similar ascertainment was applied for Parkinson’s disease, all Parkinsonian conditions (e.g., Multiple System Atrophy), and Amyotrophic Lateral Sclerosis (**sTable 45**). The first record of diagnosis was used to define date of disease onset.

**UKB covariates**

Covariates were quantified concurrently with plasma proteomic measurements. Ascertainment of hypertension and diabetes leveraged data from the GEMINI project^41^.

**Johns Hopkins Multiple Sclerosis Center (JHMSC)**

JHMSC consists of participants primarily from the metropolitan area of Baltimore, MD who attended visits at the Johns Hopkins MS Center. Blood samples were collected concurrently with outcome measurements (e.g., cognitive assessment) using standard protocols and frozen at -80°C until analysis. Participants were excluded for missing covariate data.

**JHMSC plasma proteomics**

Proteins were measured using the SomaScan v4.1 platform (7,288 SOMAmer reagents). Samples from participants that did not pass SomaScan QC criteria were excluded (n=3). Values were log_2_ transformed and those beyond 5SDs were winsorized.

**JHMSC MS diagnosis**

Relapsing-remitting and progressive MS were diagnosed based on the 2017 McDonald criteria.

**JHMSC neurological tasks**

Participants completed the 9‐Hole Peg Test, a 25‐Foot Walk Test, and a validated, iPad-based tool for screening processing speed^42^.

**JHMSC 3T MRI**

Brain MRI scans were collected on several different clinical and research scanners. Three primary whole-brain sequences without gaps were utilized: T1-weighted MPRAGE (sagittal; acquired resolution: 1 mm × 1 mm × 1 mm), T2-FLAIR (sagittal; acquired resolution: 1 mm × 1 mm × 1 mm), multi-slice T2-weighted dual-echo turbo spin echo (DE-TSE; acquired resolution: 0.8-1x 0.8-1 mm, slice thickness: 3-5 mm). The MRI images were harmonized using Harmonization with Attention-based Contrast, Anatomy, and Artifact Awareness (HACA3) to produce a consistent image contrast for downstream processing^43^. Brain substructure volumes were generated by utilizing a previously described segmentation algorithm (Multi-atlas Cortical Reconstruction using Implicit Surface Evolution; MaCRUISE)^44^. Lesion segmentation was performed using an in-house deep-learning model based on Self-Ensambled Lesion Fusion (SELF)^45^. Intracranial volume was determined via a deep neural network trained to segment the interior of the skull table^46^. All volumes were normalized to the mean intracranial volume (ICV) of the corresponding participant across all visits to account for the inter-person skull size variability. For bilateral structures, the volume fractions of the right and left sides were summed to calculate a total volume fraction, which was used for the analysis. Cortical gray matter, subcortical GM, and whole brain volume (WBV) fractions were calculated by summing the volume fractions of their components. All MRIs underwent quality control based on the following criteria: scans with ICVs below the 0.25th percentile; scans identified as outliers through PCA on the total volume matrix (specifically those exceeding ±3 SDs for the first and second principal components); scans where WBV or gray matter volume (GMV) deviated by more than ±3 SDs from the overall mean or within-person SD; and scans with more than a 10% change in WBV or GMV compared to a prior scan. No scans were excluded from the dataset, as no scans met these exclusion criteria.

**JHMSC covariates**

Covariates were quantified concurrently with plasma proteomic measurements.

**Johns Hopkins Hospital Neurology Clinic Cohort (JHN)**

JHN consists of participants primarily from the metropolitan area of Baltimore, MD who attended visits at the Johns Hopkins Hospital Cerebrospinal Fluid Center in the Department of Neurology. Blood samples were collected concurrently using standard protocols and frozen at -80°C until analysis. Participants were excluded for missing covariate data.

**JHN plasma proteomics**

Proteins were measured using the SomaScan v4.1 platform (7,288 SOMAmer reagents). CVs were calculated using QC pooled, matrix-matched sample replicates provided by SomaLogic to monitor overall assay performance. The paired plasma-CSF study design spanned 3 plates for each biological matrix (plasma or CSF) and included 3 matrix-matched QC sample replicates in each plate. CVs were calculated as CV=100*sd(RFU)/mean(RFU), where RFU is the SOMAmer intensity in Relative Fluorescence Units measured for all intra- and inter-plate QC samples. Proteins with CVs>50% were excluded (n=20); OMG’s CV was 2.5%. Samples from participants that did not pass SomaScan QC criteria and samples identified as outliers by principal component analyses were excluded (n=3). Values were log_2_ transformed and those beyond 5SDs were winsorized.

**JHN CSF proteomics**

Proteins were measured using the SomaScan v4.1 platform (7,288 SOMAmer reagents), as described above. Proteins with CVs>50% were excluded (n=7); OMG’s CV was 9.3%. Samples from participants that did not pass SomaScan QC criteria and samples identified as outliers by SomaScan or by principal component analyses were excluded (n=3). Values were log_2_ transformed and those beyond 5SDs were winsorized.

**JHN covariates**

Covariates were quantified concurrently with biofluid proteomic measurements.

**Two-sample Mendelian randomization**

CSF pQTLs were obtained from a Washington University in St. Louis GWAS of SomaScan CSF protein levels (n=3,506; 15 *cis* pQTLs at p<5.0x10^-4^)^47^. Brain pQTLs were obtained from a GWAS of dorsolateral prefrontal cortex protein levels in ROSMAP and Banner cohorts (mass spectrometry; n=716; 307 *cis* pQTLs at p<5.0×10^−8^)^48^. Plasma pQTLs were obtained from GWAS of plasma protein levels in the deCODE cohort (SomaScan; n=35,559; 406 *cis* pQTLs at p<1.8x10^-9^), the Fenland cohort (SomaScan; n=10,708; 7 *cis* pQTLs at p<1.004×10^−11^), ARIC (SomaScan; n=7,597; 10 *cis* pQTLs at p<5.0×10^−8^), and the UKB (Olink; n=54,219; 282 *cis* pQTLs at p<1.7×10^−11^)^49-52^. For CSF pQTLs, no *cis* variants (i.e., ±1 Mb) were detected at genome-wide significance (i.e., p<5.0×10^−8^); for brain and plasma pQTLs, genome-wide significance thresholds were used to detect *cis* variants. These GWAS were conducted on proteomic measurements expressed on log_10_ (WashU, CSF), log_2_ (ROSMAP/Banner, brain tissue; ARIC, plasma; UKB, plasma) or rank-based inverse normal transformation (deCODE, plasma; Fenland, plasma) scales. For outcomes, we used two GWAS summary statistics of AD from 2022 (n=487,511; AD cases=39,106; AD proxy cases=46,828)^53^ and 2019 (n=63,926; AD cases=21,982)^54^. We also used GWAS summary statistics of all-cause dementia (n=218,792; cases=5,933)^55^, vascular dementia (n=211,687; cases=387)^55^, Parkinson’s disease (n=482,730; cases=33,674)^56^, amyotrophic lateral sclerosis (n=138,086; cases=27,205)^57^, Lewy body dementia (n=6,618; cases=2,591)^58^, and multiple sclerosis (n=115,803; cases=47,429)^59^. pQTLs were pruned to remove variants in linkage disequilibrium (*r*^2^<0.05; 10 Mb window) with the 1000 Genomes Project (European) as the reference panel. Due to the availability of a single genetic instrument, the Wald ratio estimate was considered for primary analyses, and testing for heterogeneity between causal estimates and horizontal pleiotropy assumptions was not applicable.

**Proteomic biological characterization**

Several complementary bioinformatic tools were used to examine the biological relevance and functional implications of OMG and its proteomic signatures. Tissue-specific enrichment utilized published findings^28^ that mapped organ-specific proteomes using human organ bulk RNA sequencing data from the Genotype-Tissue Expression project (GTex; https://gtexportal.org/home/), where a cognate gene encoding a protein was considered enriched if it was expressed at least four times higher in a single organ compared to any other organ. Cell-specific enrichment utilized published findings^60^ that mapped cell-specific proteomes using single-cell RNA sequencing data from the Human Protein Atlas (HPA; https://www.proteinatlas.org), where a cognate gene encoding a protein was considered enriched if it was expressed at least two standard deviations higher in a single cell type compared to its relative expression across all other cell types. For assessing expression across 76 cell types and 54 tissue types, we used RNA sequencing data from the HPA; for additional assessment of expression across tissue 52 types, we used additional RNA sequencing data from GTex. RNA-seq used for differential expression analyses in post-mortem AD tissue was generated from more than 1,100 individuals (> 2,100 samples) spanning three human cohort studies, namely the ROSMAP, Mayo RNAseq, and the Mount Sinai Brain Bank. RNA-seq used for pseudo-progression analyses in post-mortem tissue was also generated from 84 individuals by the Seattle Alzheimer’s Disease Brain Cell Atlas consortium^61^. Studies were identified that externally validated the OMG SOMAmer reagent using one or more of the following techniques: multiple reaction monitoring or data dependent analysis mass spectrometry, protein quantitative trait loci (pQTLs), orthogonal strategies (e.g., ELISA), and/or proximity extension assay (i.e., Olink platform). The OpenTargets platform was used to identify existing medications with specific targets. The curated protein interaction database Human Integrated Protein-Protein Interaction rEference (HIPPIE) was used to assess evidence of protein-protein interactions. Supplementary information relevant to AD (e.g., nominated therapeutic targets, primary biologic domain etc.,) was obtained from the AD Knowledge Portal (https://adknowledgeportal.synapse.org), a platform for accessing data, analyses, and tools generated by the Accelerating Medicines Partnership Program for AD and other NIA-supported programs. Enriched biological pathways and upstream regulators were identified using Ingenuity Pathway Analysis (Qiagen Inc; version 01-22-01), a bioinformatics application that facilitates the analyses and interpretation of ‘-omics’ data using curated content available via the Ingenuity Knowledge Base (IKB). Proteins were mapped to the IKB using Entrez gene symbols of the cognate genes encoding each protein. Differential expression patterns reflected associations with OMG abundance. The user dataset was used as the reference set (i.e., the population of genes considered for *p*-value calculations was limited to those contained on the SomaScan platform), and direct as well as indirect relationships were considered. Benjamini-Hochberg (BH) FDR adjusted *p*-values derived from Fisher’s exact tests quantified the probability of overlap between cognate genes encoding proteins and molecules known to exist within a specific pathway due to random chance. Additional enrichment analyses using publicly available databases (e.g., Gene Ontology) were conducted via the Enrichr platform (https://maayanlab.cloud/Enrichr/)^62^. Similar to IPA, BH FDR adjusted *p*-values derived from Fisher’s exact tests quantified the probability of overlap between cognate genes encoding proteins and molecules known to exist within a specific term due to random chance. The combined scores reflected the product of *p*-values (log transformed) from Fisher’s exact tests multiplied by the z-scores of the deviations from each gene’s expected rank, which were derived from randomly imputed gene sets for each term. Supplementary information on individual SNPs was obtained with the OpenTargets (https://www.opentargets.org), Genecards (https://www.genecards.org), OnTime (https://ontime.wustl.edu), and GTEx platforms.

**Statistical analyses**

Statistical analyses were conducted separately in each cohort for each outcome of interest rather than conducting analyses on harmonized proteomic and outcome data across cohorts due to the inherently heterogenous study designs, data structures, and outcome distributions between cohorts. A hierarchical gating approach was employed, whereby proteins of interest for downstream analyses were first identified with discovery analyses of cortical Aβ deposition in two community-based cohorts (BLSA, ARIC). We subsequently employed multiple external cohorts and orthogonal strategies to support associations between OMG and ADRD endophenotypes (e.g., prevalent and incident cognitive impairment, biofluid biomarkers, structural neuroimaging etc.,). Covariates were selected based on theory-driven rationale and data availability. Participants with missing predictor, outcome, and/or covariate data were not included in analyses.

In the BLSA, logistic regression adjusted for age, sex, race, education, *APOE*ε4, and a comorbidity index was used to examine associations with Aβ PET and cognitive status. Multiple linear regression adjusted for the aforementioned covariates was used to examine associations with 3T MRI measures. Regional and voxel-wise brain volume analyses also adjusted for total intracranial volume, white matter volume analyses also adjusted for total white matter volume, and plasma biomarker analyses also adjusted for eGFR. Multiple linear regression adjusted for age, time between blood draw and autopsy, and most recent cognitive performance (Mini-Mental State Examination) was used to examine associations with neuropathology (Braak stages, CERAD scores) and mass spectrometry proteomics; time between blood draw and autopsy was adjusted for given the heterogeneity in duration between plasma and brain tissue sample collection dates. In ARIC, logistic regression adjusted for age, sex, race-center, education, APOEε4, and cardiovascular risk factors (obesity, diabetes, hypertension) was used to examine associations with Aβ PET status. Plasma biomarker analyses also adjusted for eGFR, smoking status, and BMI instead of obesity. Cox proportional hazards regression adjusted for the aforementioned covariates was used to examine associations with incident dementia risk. For stratified analyses, cardiovascular disease was defined as the presence of one or more conditions (congestive heart failure, stroke, diabetes, ischemic heart disease), and high education was defined as having at least some level of college. Multiple linear regression adjusted for the aforementioned covariates plus intracranial volume was used to examine associations with 3T MRI measures. In the Emory-ADRC, logistic and linear regression adjusted for age, sex, and APOEε4 were used to examine associations with cognitive status and CSF biomarkers, respectively. In the Stanford cohort, ROSMAP, Knight-ADRC, and ADNI, logistic regression adjusted for age, sex, and APOEε4 was used to examine associations with cognitive status. Cohort specific effects were assessed in fixed effect inverse variance-weighted meta-analysis for primary dichotomous outcomes of interest (i.e., Aβ PET and cognitive status) and two-sample MR outcomes. Meta-analysis of cognitive status utilized odds ratios and error estimates of OMG derived only from SomaScan platform to avoid platform-related measurement heterogeneity. Heterogeneity statistics (Q, I², t²) indicated no significant assumption violations and sensitivity analyses using random-effects models yielded similar results. In the Stanford cohort, regional brain volume analyses adjusted for age, sex, and total intracranial volume. In NILS-LSA, Cox proportional hazards regression adjusted for age, sex, and APOEε4 was used to examine associations with incident dementia risk. In Whitehall II, Cox proportional hazards and linear mixed effects regression adjusted for age, sex, and ethnicity were used to examine associations with incident dementia risk and rates of cognitive decline, respectively. In the UKB, Cox proportional hazards regression adjusted for age, sex, education, study site, APOEε4, eGFR, and cardiovascular risk factors (BMI, diabetes, hypertension, smoking) was used to examine associations with etiology-specific dementia risk. Multiple linear regression adjusted for age, sex, APOEε4, eGFR, total household income, and cardiovascular risk factors (BMI, hypertension, diabetes, smoking) was used to examine associations with 3T MRI measures. In JHMSC, logistic and linear regression adjusted for age, sex, and race were used to examine associations with MS status and MS phenotypes, respectively; regional brain volume analyses also adjusted for total intracranial volume. For plasma-CSF proteomic associations in the Knight-ADRC, the Emory-ADRC, ADNI, and JHN, linear regression adjusted for age and sex were used.

Discovery analyses of cortical Aβ deposition used a nominally significant p-value of 0.05 (although FDR-corrected results are also presented) followed by the utilization of multiple external cohorts and orthogonal strategies to reduce the possibility of Type I error. Given the targeted nature of follow up ADRD endophenotype analyses (i.e., a single protein’s measurement [OMG] in relation to specific outcomes of interest [cognitive status, ADRD biomarkers, 3T MRI etc.,]), statistical significance for these analyses was defined as p<0.05. Given the large number of comparisons when assessing OMG’s CSF, plasma, and brain proteomic signatures, statistical significance for these analyses was defined as FDR<0.05. FDR corrections were applied per outcome within each cohort. Because the multiple testing burden, power, and outcome distributions can inherently differ by study design, harmonizing statistical thresholds across heterogeneous datasets was not employed. Regression analyses leveraged several commonly employed R packages, including stats and survival. Model quality and goodness of fit were assessed using the performance R package. Meta-analysis utilized the meta R package. Model assumptions were satisfied and normal distributions were verified. Proportional hazards assumptions were assessed with Kaplin Meier curves and Schoenfeld residuals. Linear regression equations followed standard formatting (i.e., outcome ~ predictor + covariates) and did not employ random effects for any included variables. Beta coefficients, odds ratios, and hazard ratios, were reported for linear, logistic, and Cox regressions, respectively. Analyses were performed using R (versions 4.2.2-4.4.1).

**Ethics statement**

The BLSA protocol was approved by the Institutional Review Board (IRB) of the National Institute of Environmental Health Science, NIH (03AG0325) and the BLSA PET study were additionally approved by the Johns Hopkins University IRB; participants gave written informed consent prior to participation. ARIC protocols were approved by IRBs at each participating center: University of North Carolina at Chapel Hill, Chapel Hill, NC; Wake Forest University, Winston-Salem, NC; Johns Hopkins University, Baltimore, MD; University of Minnesota, Minneapolis, MN; and University of Mississippi Medical Center, Jackson, MS. ARIC participants gave written informed consent at each study visit; proxies provided consent for participants who were judged to lack capacity. Emory-ADRC participants provided informed consent under protocols approved by the IRB at Emory University. Participants (or their legally authorized representative) from the Stanford cohort provided informed consent under protocols approved by Stanford University. ROSMAP was approved by an IRB at Rush University Medical Center (L91020181, L86121802) and participants signed an informed consent, Anatomic Gift Act, and an RADC Repository consent (L99032481). The Knight-ADRC protocol was approved by the IRB at Washington University School of Medicine in St. Louis and participants provided informed consent. The IRBs of all participating ADNI institutions approved the procedures used in this study, and written informed consent was obtained from participants or surrogates. The NILS-LSA protocols were approved by the IRB of the National Center for Geriatrics and Gerontology (22TB5 and 1665-3) and all participants gave written informed consent prior to participation. Whitehall II was most recently approved by the University College London Hospital Committee on the Ethics of Human Research (85/0938); participants provided written informed consent. The UKB protocol was approved by the National Health Service National Research Ethics Service (11/NW/0382); participants gave informed consent. JHMSC protocol was approved by the Johns Hopkins University IRB; participants gave informed consent. Subjects referred to JHN consented to banking of residual CSF after clinical testing under an Johns Hopkins University IRB approved protocol.

**Data availability**

All BLSA data generated in the present study are included in this article, available on reasonable request, or in an online public repository. BLSA proteomic data are available via the Alzheimer’s Disease Data Initiative as part of the participation in the Global Neurodegeneration Proteomics Consortium (https://www.neuroproteome.org). Participants did not consent to unrestricted data sharing. Anonymized data not published within this article may be shared upon request from qualified investigators for purposes of replicating procedures and findings. Researchers who wish to use BLSA data (including proteomics) are encouraged to develop a pre-analysis plan that can be submitted for approval (https://blsa.nia.nih.gov/how-apply). ARIC proteomic data is available through the Biologic Specimen and Data Repository Information Coordinating Center (https://biolincc.nhlbi.nih.gov/studies/aric/). ARIC phenotype and proteomic data will also soon be accessible through NIH’s database of Genotypes and Phenotypes (https://www.ncbi.nlm.nih.gov/projects/gap/cgi-bin/study.cgi?study_id=phs000280.v8.p2). Additional data requests may be submitted to the ARIC steering committee (https://aric.cscc.unc.edu/aric9/researchers/Obtain_Submit_Data) and will be reviewed to ensure that data can be shared without compromising participant confidentiality or breaching intellectual property restrictions. Participant-level demographic, clinical, and proteomic data may be partially restricted based on prior participant consent, and data sharing restrictions may also be applied to ensure consistency with confidentiality or privacy laws and considerations (https://sites.cscc.unc.edu/aric/). Emory-ADRC raw data related to this manuscript are available for download (https://www.synapse.org/3platformEmory). Stanford cohort data are available upon reasonable request to the Stanford-ADRC data release committee (https://web.stanford.edu/group/adrc/cgi-bin/web-proj/datareq.php). Stanford data from specific cohorts can be requested from the following: SADRC, Tony Wyss-Coray (twc@stanford.edu); SAMS, Beth Mormino (bmormino@stanford.edu) or Anthony Wagner (awagner@stanford.edu); BPD and SCMD, Kathleen Poston (klposton@stanford.edu). ROSMAP resources are available upon request (https://www.radc.rush.edu and https://www.synpase.org). Knight-ADRC data are available upon reasonable request to The National Institute on Aging Genetics of Alzheimer’s Disease Data Storage Site (https://www.niagads.org/knight-adrc-collection). ADNI data can be requested using the ADNI database (https://adni.loni.usc.edu/). NILS-LSA data are available from the authors (iwa-waga@nec.com) upon reasonable request and with permissions of NILS-LSA investigators (https://www.ncgg.go.jp/research/lab/cgss/department/ep/index.html); as the data are under license for the current study, some restrictions to data sharing may apply. Whitehall II data requests may be submitted to the Whitehall II steering committee (whitehall2@ucl.ac.uk) and will be reviewed to ensure that data can be shared without compromising participant confidentiality or breaching intellectual property restrictions. Participant-level demographic, clinical, and proteomic data may be partially restricted based on prior participant consent, and data sharing restrictions may also be applied to ensure consistency with confidentiality or privacy laws and considerations (https://www.ucl.ac.uk/epidemiology‐health‐care/research/epidemiology‐and‐public‐health/research/whitehall‐ii/data‐sharing). Data, protocols, and other metadata of the UKB are available to the scientific community upon request in accordance with the UKB data sharing policy (https://www.ukbiobank.ac.uk/enable-your-research/apply-for-access). JHMSC data are available on reasonable request from the authors. JHN participants did not consent to unrestricted data sharing.

**References**

1. Shock, N.W.*, et al.* *Normal human aging: The Baltimore longitudinal study of aging*, (National Institutes of Health, Washington, D.C., 1984).

2. Candia, J., Daya, G.N., Tanaka, T., Ferrucci, L. & Walker, K.A. Assessment of variability in the plasma 7k SomaScan proteomics assay. *Sci Rep* **12**, 17147 (2022).

3. Bilgel, M.*, et al.* Longitudinal changes in Alzheimer's-related plasma biomarkers and brain amyloid. *Alzheimers Dement* (2023).

4. O'Brien, R.J.*, et al.* Neuropathologic studies of the Baltimore Longitudinal Study of Aging (BLSA). *J Alzheimers Dis* **18**, 665-675 (2009).

5. Hales, C.M.*, et al.* Changes in the detergent-insoluble brain proteome linked to amyloid and tau in Alzheimer's Disease progression. *Proteomics* **16**, 3042-3053 (2016).

6. Resnick, S.M., Pham, D.L., Kraut, M.A., Zonderman, A.B. & Davatzikos, C. Longitudinal magnetic resonance imaging studies of older adults: a shrinking brain. *J Neurosci* **23**, 3295-3301 (2003).

7. Duggan, M.R.*, et al.* Proteome-wide analysis identifies plasma immune regulators of amyloid-beta progression. *Brain, Behavior, and Immunity* **120**, 604-619 (2024).

8. Doshi, J.*, et al.* MUSE: MUlti-atlas region Segmentation utilizing Ensembles of registration algorithms and parameters, and locally optimal atlas selection. *Neuroimage* **127**, 186-195 (2016).

9. Davatzikos, C., Genc, A., Xu, D. & Resnick, S.M. Voxel-based morphometry using the RAVENS maps: methods and validation using simulated longitudinal atrophy. *Neuroimage* **14**, 1361-1369 (2001).

10. Lauzon, C.B.*, et al.* Simultaneous analysis and quality assurance for diffusion tensor imaging. *PLoS One* **8**, e61737 (2013).

11. Yang, Z.*, et al.* Brain aging patterns in a large and diverse cohort of 49,482 individuals. *Nature Medicine* (2024).

12. Doshi, J., Erus, G., Habes, M. & Davatzikos, C. DeepMRSeg: A convolutional deep neural network for anatomy and abnormality segmentation on MR images. *arXiv preprint arXiv:1907.02110* (2019).

13. Armstrong, N.M.*, et al.* Association of hippocampal volume polygenic predictor score with baseline and change in brain volumes and cognition among cognitively healthy older adults. *Neurobiol Aging* **94**, 81-88 (2020).

14. Duggan, M.R.*, et al.* Herpes Viruses in the Baltimore Longitudinal Study of Aging: Associations With Brain Volumes, Cognitive Performance, and Plasma Biomarkers. *Neurology*, 10.1212/WNL.0000000000201036 (2022).

15. Wright, J.D.*, et al.* The ARIC (Atherosclerosis Risk In Communities) Study: JACC Focus Seminar 3/8. *J Am Coll Cardiol* **77**, 2939-2959 (2021).

16. Walker, K.A.*, et al.* Large-scale plasma proteomic analysis identifies proteins and pathways associated with dementia risk. *Nature Aging* **1**, 473-489 (2021).

17. Lu, Y.*, et al.* Changes in Alzheimer Disease Blood Biomarkers and Associations With Incident All-Cause Dementia. *Jama* **332**, 1258-1269 (2024).

18. Gottesman, R.F.*, et al.* The ARIC-PET amyloid imaging study: Brain amyloid differences by age, race, sex, and APOE. *Neurology* **87**, 473-480 (2016).

19. Walker, K.A.*, et al.* Proteomics analysis of plasma from middle-aged adults identifies protein markers of dementia risk in later life. *Sci Transl Med* **15**, eadf5681 (2023).

20. Gottesman, R.F.*, et al.* Associations Between Midlife Vascular Risk Factors and 25-Year Incident Dementia in the Atherosclerosis Risk in Communities (ARIC) Cohort. *JAMA Neurol* **74**, 1246-1254 (2017).

21. Knopman, D.S.*, et al.* Mild Cognitive Impairment and Dementia Prevalence: The Atherosclerosis Risk in Communities Neurocognitive Study (ARIC-NCS). *Alzheimers Dement (Amst)* **2**, 1-11 (2016).

22. Schneider, A.L.C.*, et al.* Diabetes, Prediabetes, and Brain Volumes and Subclinical Cerebrovascular Disease on MRI: The Atherosclerosis Risk in Communities Neurocognitive Study (ARIC-NCS). *Diabetes Care* **40**, 1514-1521 (2017).

23. Knopman, D.S.*, et al.* Vascular imaging abnormalities and cognition: mediation by cortical volume in nondemented individuals: atherosclerosis risk in communities-neurocognitive study. *Stroke* **46**, 433-440 (2015).

24. Power, M.C.*, et al.* Midlife and late‐life vascular risk factors and white matter microstructural integrity: the atherosclerosis risk in communities neurocognitive study. *Journal of the American Heart Association* **6**, e005608 (2017).

25. Raz, L.*, et al.* Thrombogenic microvesicles and white matter hyperintensities in postmenopausal women. *Neurology* **80**, 911-918 (2013).

26. Dammer, E.B.*, et al.* Multi-platform proteomic analysis of Alzheimer's disease cerebrospinal fluid and plasma reveals network biomarkers associated with proteostasis and the matrisome. *Alzheimers Res Ther* **14**, 174 (2022).

27. Olsson, A.*, et al.* Simultaneous Measurement of β-Amyloid(1–42), Total Tau, and Phosphorylated Tau (Thr181) in Cerebrospinal Fluid by the xMAP Technology. *Clinical Chemistry* **51**, 336-345 (2005).

28. Oh, H.S.-H.*, et al.* Organ aging signatures in the plasma proteome track health and disease. *Nature* **624**, 164-172 (2023).

29. Oh, H.S.-H.*, et al.* Synapse protein signatures in cerebrospinal fluid and plasma predict cognitive maintenance versus decline in Alzheimer’s disease. *bioRxiv*, 2024.2007.2022.604680 (2024).

30. Bennett, D.A.*, et al.* Religious Orders Study and Rush Memory and Aging Project. *J Alzheimers Dis* **64**, S161-s189 (2018).

31. Bennett, D.A.*, et al.* Decision rules guiding the clinical diagnosis of Alzheimer's disease in two community-based cohort studies compared to standard practice in a clinic-based cohort study. *Neuroepidemiology* **27**, 169-176 (2006).

32. Fernandez, M.V.*, et al.* Genetic and multi-omic resources for Alzheimer disease and related dementia from the Knight Alzheimer Disease Research Center. *Scientific Data* **11**, 768 (2024).

33. Yang, C.*, et al.* Genomic atlas of the proteome from brain, CSF and plasma prioritizes proteins implicated in neurological disorders. *Nat Neurosci* **24**, 1302-1312 (2021).

34. Duggan, M.R.*, et al.* Proteomic analyses reveal plasma EFEMP1 and CXCL12 as biomarkers and determinants of neurodegeneration. *Alzheimer's & Dementia*.

35. Otsuka, R.*, et al.* Dietary diversity and risk of late-life disabling dementia in middle-aged and older adults. *Clinical Nutrition* **42**, 541-549 (2023).

36. Marmot, M.G.*, et al.* Health inequalities among British civil servants: the Whitehall II study. *Lancet* **337**, 1387-1393 (1991).

37. Williams, S.A.*, et al.* Plasma protein patterns as comprehensive indicators of health. *Nat Med* **25**, 1851-1857 (2019).

38. Lindbohm, J.V.*, et al.* Plasma proteins, cognitive decline, and 20-year risk of dementia in the Whitehall II and Atherosclerosis Risk in Communities studies. *Alzheimers Dement* **18**, 612-624 (2022).

39. Eldjarn, G.H.*, et al.* Large-scale plasma proteomics comparisons through genetics and disease associations. *Nature* **622**, 348-358 (2023).

40. Alfaro-Almagro, F.*, et al.* Image processing and Quality Control for the first 10,000 brain imaging datasets from UK Biobank. *Neuroimage* **166**, 400-424 (2018).

41. Murrin, O.*, et al.* A systematic analysis of the contribution of genetics to multimorbidity and comparisons with primary care data. *medRxiv*, 2024.2005.2013.24307009 (2024).

42. Siavoshi, F., Ladakis, D.C., Muller, A., Nourbakhsh, B. & Bhargava, P. Ocrelizumab alters the circulating metabolome in people with relapsing-remitting multiple sclerosis. *Ann Clin Transl Neurol* **11**, 2485-2498 (2024).

43. Zuo, L.*, et al.* HACA3: A unified approach for multi-site MR image harmonization. *Computerized Medical Imaging and Graphics* **109**, 102285 (2023).

44. Huo, Y.*, et al.* Consistent cortical reconstruction and multi-atlas brain segmentation. *Neuroimage* **138**, 197-210 (2016).

45. Zhang, J.*, et al.* Towards an Accurate and Generalizable Multiple Sclerosis Lesion Segmentation Model Using Self-Ensembled Lesion Fusion. in *2024 IEEE International Symposium on Biomedical Imaging (ISBI)* 1-5 (2024).

46. Singh, M.*, et al.* Accurate Estimation of Total Intracranial Volume in MRI using a Multi-tasked Image-to-Image Translation Network. *Proc SPIE Int Soc Opt Eng* **11596**(2021).

47. Western, D.*, et al.* Proteogenomic analysis of human cerebrospinal fluid identifies neurologically relevant regulation and implicates causal proteins for Alzheimer’s disease. *Nature Genetics* (2024).

48. Wingo, A.P.*, et al.* Sex differences in brain protein expression and disease. *Nature Medicine* **29**, 2224-2232 (2023).

49. Sun, B.B.*, et al.* Plasma proteomic associations with genetics and health in the UK Biobank. *Nature* **622**, 329-338 (2023).

50. Ferkingstad, E.*, et al.* Large-scale integration of the plasma proteome with genetics and disease. *Nat Genet* **53**, 1712-1721 (2021).

51. Pietzner, M.*, et al.* Mapping the proteo-genomic convergence of human diseases. *Science* **374**, eabj1541 (2021).

52. Zhang, J.*, et al.* Plasma proteome analyses in individuals of European and African ancestry identify cis-pQTLs and models for proteome-wide association studies. *Nat Genet* **54**, 593-602 (2022).

53. Bellenguez, C.*, et al.* New insights into the genetic etiology of Alzheimer's disease and related dementias. *Nat Genet* **54**, 412-436 (2022).

54. Kunkle, B.W.*, et al.* Genetic meta-analysis of diagnosed Alzheimer's disease identifies new risk loci and implicates Aβ, tau, immunity and lipid processing. *Nat Genet* **51**, 414-430 (2019).

55. Kurki, M.I.*, et al.* FinnGen provides genetic insights from a well-phenotyped isolated population. *Nature* **613**, 508-518 (2023).

56. Nalls, M.A.*, et al.* Identification of novel risk loci, causal insights, and heritable risk for Parkinson's disease: a meta-analysis of genome-wide association studies. *Lancet Neurol* **18**, 1091-1102 (2019).

57. van Rheenen, W.*, et al.* Common and rare variant association analyses in amyotrophic lateral sclerosis identify 15 risk loci with distinct genetic architectures and neuron-specific biology. *Nat Genet* **53**, 1636-1648 (2021).

58. Chia, R.*, et al.* Genome sequencing analysis identifies new loci associated with Lewy body dementia and provides insights into its genetic architecture. *Nat Genet* **53**, 294-303 (2021).

59. Multiple sclerosis genomic map implicates peripheral immune cells and microglia in susceptibility. *Science* **365**(2019).

60. Cordon, J.*, et al.* Identification of Clinically Relevant Brain Endothelial Cell Biomarkers in Plasma. *Stroke* **54**, 2853-2863 (2023).

61. Gabitto, M.I.*, et al.* Integrated multimodal cell atlas of Alzheimer’s disease. *Nature Neuroscience* **27**, 2366-2383 (2024).

62. Chen, E.Y.*, et al.* Enrichr: interactive and collaborative HTML5 gene list enrichment analysis tool. *BMC Bioinformatics* **14**, 128 (2013).
